# Reactions of an Anionic Gallylene with Azobenzene or Azide Compounds Through C(sp^2^)–H and C(sp^3^)–H Activation

**DOI:** 10.3390/molecules29215021

**Published:** 2024-10-24

**Authors:** Jinfeng Sun, Fangfeng Chen, Juan Liu, Yihu Zhang, Dongyu He, Vladimir A. Dodonov, Yanxia Zhao

**Affiliations:** 1 Key Laboratory of Synthetic and Natural Functional Molecule of the Ministry of Education, College of Chemistry and Materials Science, Northwest University, Xi’an 710069, China; 2 Grigory Alekseevich Razuvaev Institute of Organometallic Chemistry of Russian Academy of Sciences (IOMC RAS), Tropinina 49, Nizhny Novgorod 603950, Russia

**Keywords:** C–H activation, gallylene, azobenzene, organic azides, α-diimine ligands

## Abstract

The activation of inert C–H bonds remains a challenge in current chemistry. Here, we report the excellent reactivity of the anionic gallylene species [LGa:][Na(THF)_3_] (L = [(2,6-*i*Pr_2_C_6_H_3_)NC(CH_3_)]_2_^2−^, **1**) that allows the selective activation one *ortho* sp^2^ C–H bond of several azobenzene and azide derivatives at ambient temperature, with the transfer of the hydrogen atom to one of the nitrogen atoms. The process leads to the formation of the aryl amido products [LGa-κ^2^N,C-PhNN(H)(*p*-R-C_6_H_3_)][Na(solvent)_3_] (**2**, R = H solvent = DME (1,2-Dimethoxyethane); **3**, R = –OMe, solvent = DME; **4**, R = –NMe_2_ solvent = THF), [LGa-κ^2^N,C-(*m*-CH_3_-C_6_H_4_)NN(H)(*m*-CH_3_-C_6_H_3_)][Na(15-C-5)_2_] (**5**) with new Ga–C and Ga–N bonds. Moreover, **1** is also effective for the C–H activation of two azides RN_3_ (R = 2,4,6-Me_3_C_6_H_2_ or 2,6-*i*Pr_2_C_6_H_3_), resulting in the formation of gallium amides [LGa(NH-2-(CH_2_)-4,6-Me_2_C_6_H_2_)][Na(15-C-5)_2_] (**6**) and [LGa(NH-2,6-*i*Pr_2_C_6_H_3_)_2_][Na(THF)_5_] (**7**) through intra- or intermolecular sp^3^ C–H amination. Significantly, these reactions occur for the highly challenging activation of inert C(sp^2^)–H and C(sp^3^)–H bonds, thus demonstrating the excellent reactivity of the Ga(I) species **1**. The products **2**–**7** were characterized by X-ray crystallography, ^1^H and ^13^C NMR, UV–vis spectroscopy, and density functional theory (DFT) calculations.

## 1. Introduction

The activation of C–H bonds has evolved into a well-established synthetic methodology for the construction of new C–C and C–Heteroatom bonds [1,2]. Traditionally, the field of C–H activation has predominantly focused on transition metals due to their characteristic partially occupied valence *d* orbitals [3,4,5,6]. Over the past decade, it has become increasingly apparent that low-valent main group metal complexes, owing to the imposed variable oxidation states of the metal, can exhibit transition-metal-like behavior in bond activation despite their lack of valence d orbitals [7]. This property, together with their ability to overcome the high cost and toxicity associated with common transition metal catalysts, makes them a promising alternative for small molecule activation [8]. However, compared to transition metals, examples of direct reactions involving stable main group elements in C–H bond activation still remain limited.

Low-valent group 13 element species have shown excellent reactivities towards various molecules, including activation of C–H bonds. In particular, the highly reactive, nucleophilic anionic Al(I) species can activate C–H bonds efficiently, although frequently in conjunction with elevated temperature, prolonged reaction duration, or catalysts.

**Scheme 1:**
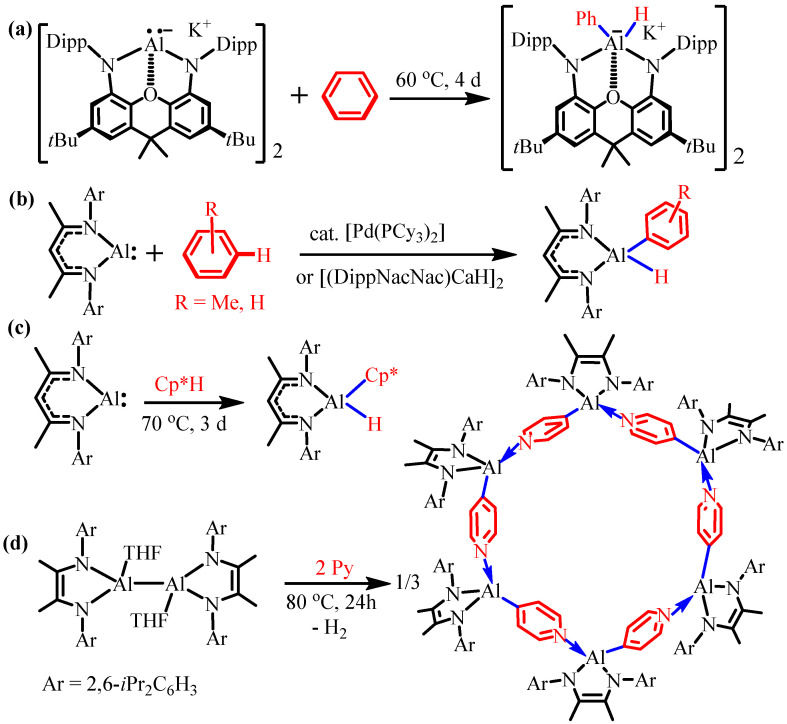
Examples of C−H bond activation at low-valent Al(II/I) centers. The blue bonds represent newly formed chemical bonds. (**a**) C–H activation of benzene by aluminyl anion [K{Al(NON)}]_2_. (**b**,**c**) C–H activation of unactivated arenes (benzene, toluene, xylenes) and Cp*H by [(^Dipp^NacNac)]Al(I). (**d**) C–H activation of pyridine by [LAl(THF)]_2_.

The Aldridge and Goicoechea group reported the oxidative addition of a benzene C–H bond using the first anionic Al(I) complex [Al(NON)]^−^ (NON = 4,5-bis(2,6-di-*i*Pr-anilido)-2,7-di-*t*Bu-9,9-di-Me-xanthene) (Scheme 1a) [9]. Crimmin and colleagues reported the oxidative addition of low-valent β-diketiminate Al(I) complex ^Dipp^NacNacAl (^Dipp^NacNac = [ArNC(Me)CHC(Me)-NAr]^−^, (Ar = 2,6-*i*Pr_2_C_6_H_3_)) with C–H bonds of toluene, benzene, and xylenes but only in the presence of a palladium catalyst (Scheme 1b) [10]. In a similar reaction, Harder et al. successfully achieved the oxidative addition of sp^2^ C–H bond on non-activated arenes (toluene, benzene, and xylene) to [(^Dipp^NacNac)]Al(I) at ambient temperature through the catalytic presence of [(^Dipp^NacNac)CaH]_2_ (Scheme 1b) [11]. The Nikonov group reported the oxidative addition of a C–H bond in sterically hindered pentamethylcyclopentadiene under controlled conditions at 70 °C for 3 days, leading to the formation of the alkyl hydride derivative of the four-coordinate aluminum [^Dipp^NacNacAlH(C_5_Me_5_)] (Scheme 1c) [12]. In 2021, our group reported a case of dialumane-mediated C–H activation of pyridines to form a unique metallo-macrocycle compound (Scheme 1d) [13].

**Scheme 2:**
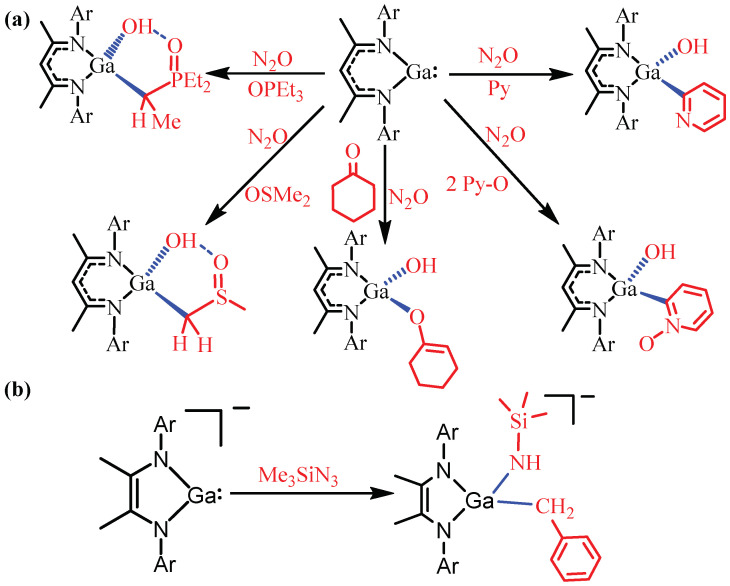
Examples of C–H bond activation at low-valent Ga(I) centers. The blue bonds represent newly formed chemical bonds. (**a**) Sequential oxidation/C–H activation on ^Dipp^NacNacGa(I). (**b**) C–H activation of mrthyl in toluene by gallylene species [LGa:][Na(THF)_3_] (**1**).

In contrast to Al(I) species, investigations on C–H bond activation by low-valent Ga compounds under ambient conditions are relatively rare. The Nikonov group reported that the oxidation of a Ga(I) compound, ^Dipp^NacNacGa(I), to transient gallium oxide or imide ^Dipp^NacNacGa = X (X = O or NR) can facilitate the addition to non-activated C–H bonds in both aromatic and aliphatic substrates, resulting in the formation of alkyl(aryl) hydroxides of gallium (Scheme 2a) [14,15,16]. Schulz et al. reported that the reactions of gallium phosphinidene with acetone or acetophenone proceeded via C(sp^3^)–H bond activation, leading to the enolization of ketones [17]. Recently, we set out to investigate the reactions of the anionic gallylene species [LGa:]^−^ (**1**) with organic azides. It was found that in the reaction with TMSN_3_, compound **1** activated a C–H bond of the methyl group on toluene through a transient anionic Ga=N species to produce a benzylic amido complex (Scheme 2b) [18]. To further explore the C–H bond activation ability of this gallylene, likely through the Ga=N imido intermediate, we carried out the reactions of **1** with azobenzene derivatives and bulky azides. Remarkably, both C(sp^2^)–H and C(sp^3^)–H bond activation processes were observed at ambient temperature, affording the products **2**–**7**. Herein, the reaction processes and characterization of the products are presented.

## 2. Results and Discussion

### 2.1. Synthesis of Complexes ***2***–***5***

**Scheme 3:**
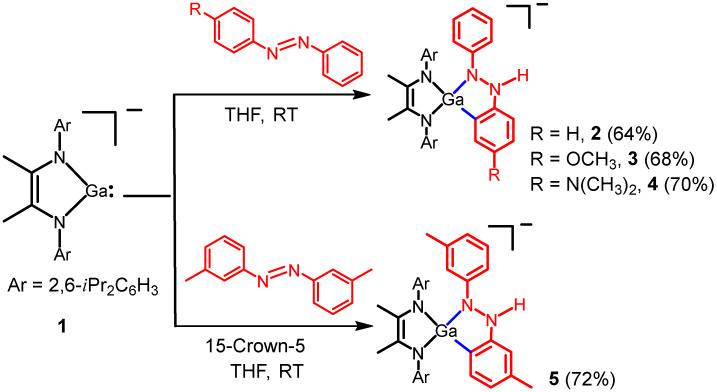
Reaction of **1** with azobenzene derivatives to yield complexes **2**–**5**. The blue bonds represent newly formed chemical bonds.

The reaction of the gallylene species **1** with azobenzene PhN=NPh resulted in the formation of an anionic complex [LGa-κ^2^N,C-PhNN(H)(C_6_H_4_)][Na(DME)_3_] (**2**, 64% yield), through activation of one *ortho*-position C–H bond in a phenyl group of azobenzene and subsequent transfer of this hydrogen to the nitrogen atom to which this phenyl ring is attached. Similarly, the reactions of **1** with azo compounds containing electron-donating groups, 4-methoxyazobenzene, 4-dimethylaminoazobenzene, and 3,3′-dimethylazobenzene also led to selective *o*-C–H activation of the phenyl ring bearing the electron-donating group, resulting in the formation of analogous complexes [LGa–κ^2^N,C–PhNN(H)(*p*-OCH_3_–C_6_H_3_)][Na(DME)_3_] (**3**, 68%), [LGa–κ^2^N,C–PhNN(H)(*p*-N(CH_3_)_2_–C_6_H_3_)][Na(THF)_3_] (**4**, 70%) and [LGa-κ^2^N,C– (*m*-CH_3_–C_6_H_4_)NN(H)(*m*-CH_3_–C_6_H_3_)][Na(15-C-5)_2_] (**5**, 72%, Scheme 3). It is noticeable that in the above complexes **2**–**5**, the azobenzene substrate, either without substituents or with electron-donating groups, underwent *o*-C–H bond cleavage and H transfer to the N atom on the same side. For further validation of this selective C–H activation, mono-acetyl and trifluoromethyl-substituted azobenzene substrates were tested. However, conducting the reaction at an elevated temperature of 80 °C did not yield the desired product, possibly due to the diminished reactivity resulting from strong electron-withdrawing effects.

**Figure 1:**
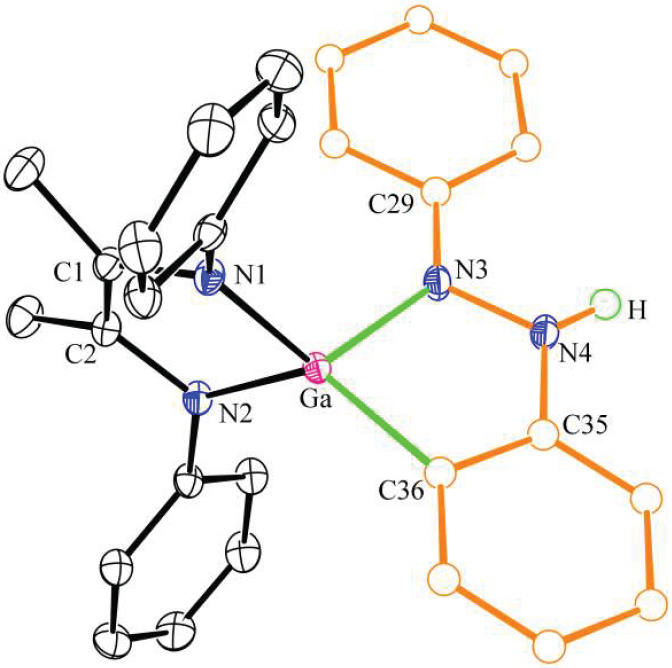
Molecular structure of **2**. Thermal ellipsoid plot (30%). H atoms except N4–H, Na(DME)_3_ counterion, and *i*Pr groups on the ligands are omitted for clarity. The green bonds represent newly formed chemical bonds.

**Figure 2:**
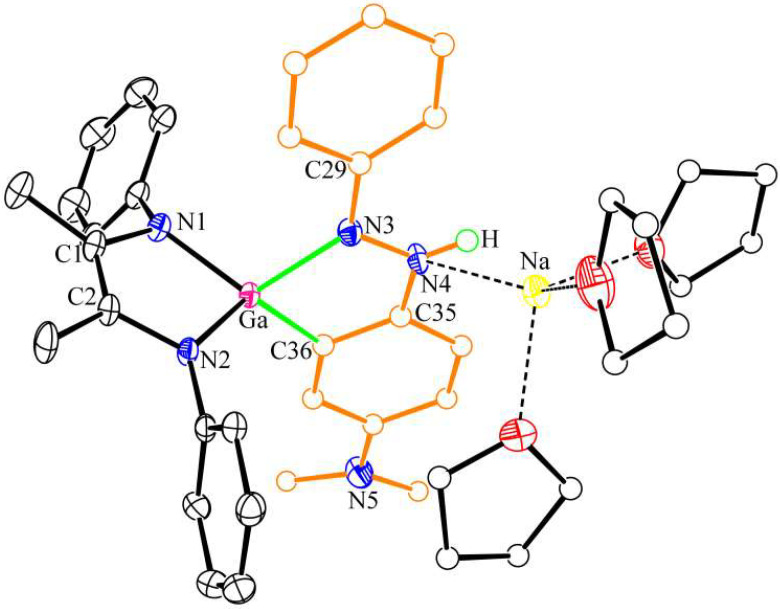
Molecular structure of **4**. Thermal ellipsoid plot (30%). H atoms except N4–H and *i*Pr groups on the ligands are omitted for clarity. The green bonds represent newly formed chemical bonds.

**Figure 3:**
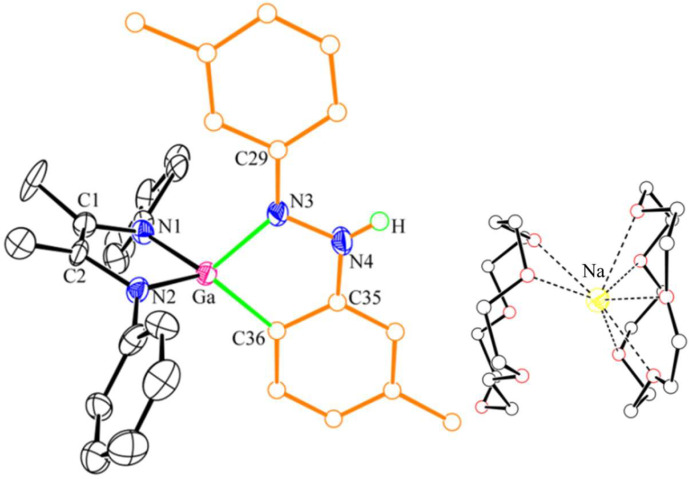
Molecular structure of **5**. Thermal ellipsoid plot (30%). H atoms except N4–H and *i*Pr groups on the ligands are omitted for clarity. The green bonds represent newly formed chemical bonds.

Complexes **2**–**5** were crystallized by slow evaporation of a THF/DME solution at ambient temperature. The crystal data and structure determination details can be found in Tables S1 and S2, while the selected bond lengths and angles are provided in Table 1. These complexes contain a monoanionic gallium moiety [LGa-κ^2^N,C–ArNN(H)Ar]^−^ and one Na^+^ counter-cation in different bonding modes. In the structures of **2–5**, upon C–H activation and hydrogen transfer, the azo compound changed to an amido-amino aryl moiety, which bonds to the gallium atom through the amido and *ortho*-carbanion groups to constitute a five-membered metallocycle (Figure 1, Figure 2 and Figure 3 and Supplementary Materials, Figure S19). Thus, together with a chelating α-diimine ligand, the Ga center resides in a distorted tetrahedral geometry containing two five-membered coordination cycles.

Azobenzene is known to be a promising building block for photo-switches owing to its facile reversible cis-trans isomerization upon irradiation/heating. Thus, the efficient and direct synthesis of intricate polysubstituted azobenzenes has garnered significant attention [19,20]. In this respect, the regio- and chemoselective activation of C–H bonds by transition metals has been an attractive and challenging strategy for synthesizing sterically constrained azo compounds [21,22]. Azo ligands, known for their redox non-innocent character, have found extensive applications in coordination and organometallic chemistry [22]. The metal complexes containing azo ligands, like azobenzene, 2,2′-azobispyridine, and phenylazopyridine, have been extensively studied [19,23,24]. In most cases, azo ligands exhibit a significant double-bond character between the nitrogen atoms, as well as a dative M←:N bond with weak intramolecular interaction between the metal center and N atoms [22]. Conversely, the reduction of the N=N double bond to a single bond is seldom observed. Jones and Murphy reported a reaction of the gallylene [:Ga{[N(Ar)C(H)]_2_}][K(tmeda)] with PhN=NPh to produce a doubly reduced product [{κ^2^N,C–PhNN(H)(C_6_H_4_)}Ga{[N(Ar)C(H)]_2_}] through C–H bond activation in azobenzene [25]. Roesky and Stalke reported that the activation of the *o*-C–H moiety in azobenzene occurred through the reaction with silylene L^1^SiNPh_2_ (L^1^ = PhC(N*t*Bu_2_) [26].

In complexes **2**−**5**, both of the GaC_2_N_2_ chelating rings exhibit a high degree of planarity, with minimal deviations (Δ = 0.142–0.152 Å) from the least-squares plane. The Ga–amide bond lengths (Ga–N3, 1.959(5)–2.026(1) Å) within the coordinated azo moiety are slightly longer compared to other related gallium amides [15,25] and are significantly longer than those to the α-diimine ligands (1.872(8)–1.911(2) Å). The lengths of the new Ga–C bonds formed through *o*-C–H bond activation of the phenyl group of azobenzene (1.916(1)−1.967(3) Å) closely resemble the Ga–C bonds found in alkyl or aryl derivatives of NacNacGa (1.9903(17)–2.0117(15) Å) [15]. The amido–amino N3–N4 bond lengths (1.426(4), 1.374(12), 1.354(4), and 1.417(8) Å in compounds **2**–**5**, respectively) in the transformed azo moiety exhibit significant elongation compared to the typical free azo N=N double bond (ca. 1.24 Å) [23]. Instead, they are closer to N–N single bond distances (ca. 1.45 Å) [27]. The C(Ph)–N bond distances (1.355(1)–1.410(7) Å) in the compounds are notably shorter compared to those observed in diazobenzene (1.43 Å).

The hydrogen transfer to the nitrogen atom was evidenced by the X-ray diffraction data and ^1^H NMR and IR spectra. The difference map reveals a significant residual electron density in the vicinity of N4 in the azobenzene fragment, indicating the presence of a hydrogen atom bonded to nitrogen. This was also confirmed by the appearance of a distinct N–H proton resonance at *δ* = 5.52, 5.18, 5.12, and 5.51 ppm in the ^1^H NMR spectra. Meanwhile, IR spectra exhibit absorption bands at 3338, 3252, 3380, and 3235 cm^−1^, corresponding to the N–H stretch for compounds **2**–**5**, respectively. The ^1^H NMR spectrums of crystals from **2**–**5** show an asymmetry in the α-diimine ligands. The methyl protons within the *i*Pr groups exhibit four doublets, while the methine protons of the *i*Pr moiety display two septets. It is worth noting that compound **2** exhibits an additional set of similar ^1^H NMR signals with the product (Figure S1). These signals have slightly different chemical shifts and coupling constants compared to the product, but their integrals and peak shapes are similar to the product.

**Scheme 4:**
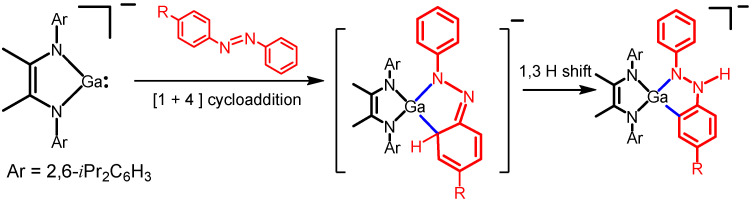
Possible mechanism for the reaction of gallylenes with azobenzene. The blue bonds represent newly formed chemical bonds.

We speculated that this could potentially serve as an intermediate in the reaction process. A plausible mechanism may involve a [1 + 4] cycloaddition of the Ga(I) center of compound **1** with azobenzene to afford the [LGa-κ^2^N,C–PhNN(C_6_H_5_)] intermediate, followed by a 1,3-migration of the ortho-aryl proton to the nitrogen center (Scheme 4). Additionally, distinct signals at 2.72 and 5.67 are observed, corresponding to the hydrogen chemical shift in Ga–C*H* and Ga–CH–C*H*-moieties within the isomer. Notably, these specific signals were absent in compounds **3**−**5**; however, attempts to isolate the intermediate have thus far been unsuccessful. A precedent for this mechanism is established by the reaction of [:Ga{[N(Ar)C(H)]_2_}][K(tmeda)] with azobenzene, leading to the formation of the doubly reduced product [{κ^2^N,C–PhNN(H)(C_6_H_4_)}Ga{[N(Ar)C(H)]_2_}] [25]. The isomer ratio, as determined from the NMR spectrum (Figure S1), is approximately 3:1 (product **2**/intermediate). In the ^1^H NMR spectrum, no isomers with electron-donating groups were observed in compound **3** (or in compounds **4** and **5**). On the basis of these results, we propose that small molecules containing electron-donating groups are more prone to undergo reactions analogous to electrophilic substitution, directly converting unstable intermediates into products. Conversely, in the presence of electron-withdrawing groups on the azobenzene, such as mono-acetyl and trifluoromethyl-substituted azobenzene, the formation of corresponding products was not observed. The IR spectrum of the absorption at 1575–1630 cm^−1^ [28], corresponding to the N=N stretching mode, disappeared. The UV–vis spectrum of complex **2**–**5** was recorded in THF. The absorption bands at wavelengths of 268–358 nm are ascribed to the π–π* transition from the dianionic α-diimine ligand (Figures S13–S18 in Supplementary Materials). Moreover, the location and coordination mode of the Na^+^ counter-cation are different in these complexes. In **2** and **3**, the Na^+^ ion is not bonded to the main structural motif but is solvated by three DME molecules. In contrast, in compound **4**, the sodium ion is coordinated with the N4 atom of the azo ligand and with three THF molecules. In the case of complex **5**, the Na^+^ ion is trapped by two 15–Crown–5 molecules to form a sandwich structure.

To gain a more comprehensive understanding of the electronic structures of complexes **2**–**5**, DFT studies at the B3LYP level with the 6–31G* basis were conducted employing model compounds **2H**–**5H**, wherein the 2,6-diisopropyl groups on the phenyls in ligand L were substituted with hydrogen atoms (for details, see Supplementary Materials, Figure S20, and Tables S3 and S4). The originally N=N double bond now exhibits a bond order of approximately 1.00, which is consistent with a reduction resulting in an N–N single bond. The Ga–N3(amide) bond exhibits an average bond order of 0.52, indicating the formation of a covalent Ga–N bond. This value is somewhat higher than the calculated average bond order of 0.44 for the Ga–N bonds to the α-diimine ligand, consistent with the presence of covalency in the latter case. According to natural population analysis (NPA), the negative partial charges observed on ligand L in **2H**–**5H** (average −1.39 e) are almost identical to those in the precursor **1** (−1.40 e), while the charges on gallium (average 1.66 e) in **2H**–**5H** are significantly higher than those observed in **1** (0.49 e). These results demonstrate that the original Ga(I) center participated in the 2e-reduction of azobenzene, while the α-diimine ligand remained unchanged. Accordingly, the entire azobenzene fragment exhibits negative partial charges of approximately −1.26 e, corresponding to the dianion character of the amido aryl moiety.

### 2.2. Synthesis of Complexes ***6*** and ***7*** from the Reactions of 1 with AZIDES

In recent decades, a wide range of main group complexes with distinctive bonding and structural characteristics have been synthesized and extensively investigated for their reactivity [29,30,31,32,33]. The reactions of organic azides with main group species provide a direct synthetic approach for N-containing main group organometallic compounds, encompassing multiple bonds and heterocycles [34]. The carbene analogs [LE:] (E = main group 13 and 14 elements) have been found to react with azides to form E=N imido species, some of which then combine with a second azide via [2 + 3] cycloaddition to produce tetrazole heterocycles [35,36,37,38,39,40,41]. Alternatively, the highly polarized E=N double bond in the imido can also break non-activated C−H bonds in aromatic and aliphatic substrates [15,35,39,42,43]. Regioselective intramolecular C(sp^3^)–H amination has been facilitated by the development of various metal-based catalytic systems, enabling the synthesis of N-heterocycles with diverse ring sizes and functional groups through a radical-type pathway [44,45,46].

We previously reported the use of gallylene species [LGa][Na(THF)_3_] (**1**) in a reaction with organic azides as a promising approach to synthesizing gallium imides. However, treatment of **1** with benzylazide (benzyl-N_3_) or trimethylsilyl azide (Me_3_SiN_3_) in toluene led to the formation of anionic, dimeric tetrazole [LGa(benzyl–N_4_–benzyl)]_2_[Na(THF)]_2_ or benzylic amido complex [LGa(NHSiMe_3_)(benzyl)][Na(THF)_4_], respectively [18]. Building upon the existing literature, our study aims to investigate the reactivity of transient gallium imide [LGa=NR]^−^ towards inert C–H bonds. In this study, we report the reaction of **1** with 2,4,6-Me_3_C_6_H_2_N_3_ or 2,6-*i*Pr_2_C_6_H_3_N_3_ and demonstrate in situ intramolecular or intermolecular C–H amination at the emerging Ga=N moiety.

**Scheme 5:**
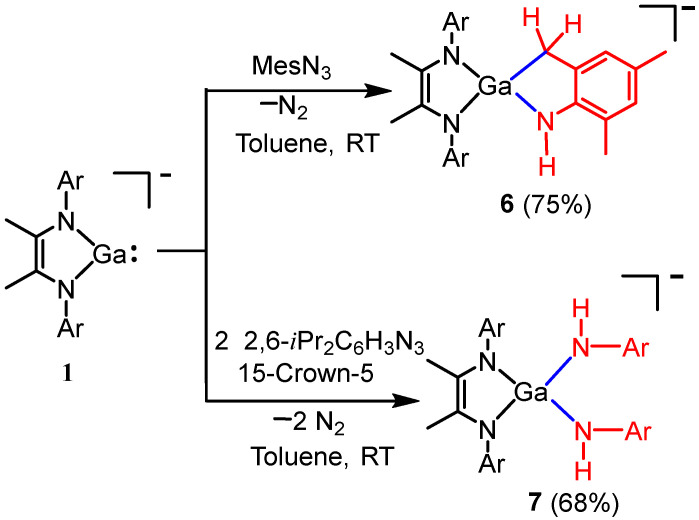
Reaction of **1** with 2,4,6-trimethylphenylN_3_ and 2,6-*i*Pr_2_C_6_H_3_N_3_ to yield **6** and **7**. The blue bonds represent newly formed chemical bonds.

The reaction between gallylene **1** and 1.0 equivalent 2,4,6-Me_3_C_6_H_2_N_3_ resulted in intramolecular C–H activation on one methyl group of the azide 2,4,6-Me_3_C_6_H_2_N_3_ at ambient temperature, forming the complex [LGa(NH-2-(CH_2_)-4,6-Me_2_C_6_H_2_)][Na(15-C-5)_2_] (**6**, 75% yield, Scheme 5). The reaction is postulated to proceed through a gallium imide LGa=NMes intermediate that undergoes C–H activation and addition of the hydrogen to the nitrene, similar with the previously documented C–H activation on a transient gallium imide [18,35]. The reaction of gallylene **1** with 2.0 equivalent MesN_3_ was also investigated, exhibiting similar reaction phenomena. The solution gradually transitions from an orange-red to a brownish-red color, accompanied by the release of nitrogen; however, isolation of the corresponding product has not been achieved. The reaction product, [LGa(NH-2-(CH_2_)-4,6-Me_2_C_6_H_2_)][Na(15–C–5)_2_] (**6**), was characterized using ^1^H and ^13^C NMR spectroscopy, while its structure was confirmed through single-crystal X-ray diffraction analysis. The ^1^H NMR spectrum of yellow crystals of **6** shows an asymmetry in the α-diimine ligands. The methyl protons within the *i*Pr groups exhibit three doublets, while the methine protons of the *i*Pr moiety display two septets (*δ*_H_ 3.87 and 4.19, 4H, C*H*(CH_3_)_2_). The protons of the (CH_3_)_2_C_2_ backbone in **6** exhibit a singlet signal (*δ*_H_ 1.51, 6H, (C*H*_3_)_2_C_2_). A new peak at 3.21 ppm is attributed to the presence of an NH moiety, while the IR spectrum indicates the occurrence of the N–H stretching vibration (*ν*_NH_ = 3499 cm^−1^).

The polarized M=N bond (M = Al, In) in the transient imide has been reported to undergo further C–H activation across the M=N bond, either through interaction with the supporting ligand [35,42,43] or via hydrogen atom abstraction from a methyl in one molecule of toluene solvent [39]. Reaction of NacNacGa and organic azide N_3_SiMe_3_ leads to the formation of a imide NacNacGa=NSiMe_3_ intermediate, which exhibits the ability to cleave unreactive sp^3^ and sp^2^ C–H bonds in various substrates (pyridine, cyclohexanone, ethyl acetate, DMSO, and OPEt_3_), resulting in the generation of gallium aryl/alkyl amides [(NacNac)GaR(NHSiMe_3_)] [15]. However, the intramolecular C–H activation has not been observed for Ga species.

**Figure 4:**
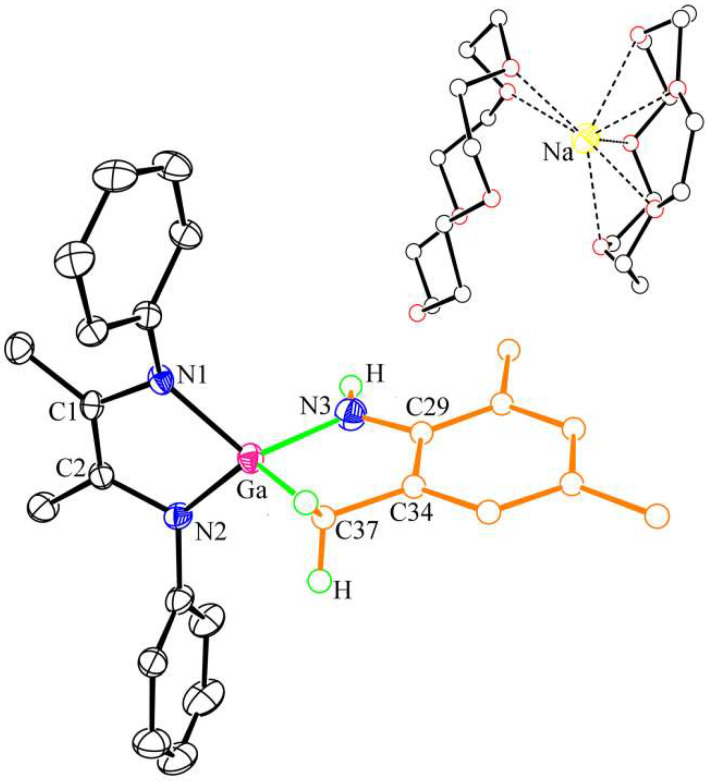
Molecular structure of **6**. Hydrogen atoms and *i*Pr groups have been omitted. The green bonds represent newly formed chemical bonds. Selected bond lengths [Å] and angles [°]: Ga–N1 1.913(4), Ga–N2 1.912(4), N–C1 1.419(5), N2–C2 1.428(6), C1–C2 1.356(6), Ga–N3 1.929(4), Ga–C37 1.995(5), N3–C29 1.372(6), C29–C34 1.407(7), C34–C37 1.506(7), N3–H 0.880, N1-Ga-N3 116.06(17), N1-Ga-C37 128.6(2), N2-Ga-N1 86.86(16), N2-Ga-N3 118.46(17), N2-Ga-C37 122.73(19), N3-Ga-C37 87.6(2).

X-ray diffraction analysis of **6** revealed a κ^2^-C,N–[NH-2-(CH_2_)-4,6-Me_2_C_6_H_2_] ligand chelating to a four-coordinate gallium (III) center (Figure 4). The sodium counter cation is trapped within two 15-Crown-5 molecules through five and two oxygen atoms, respectively. The Ga center connects two fused rings (GaC_2_N_2_ and GaC_3_N) arranged in an almost orthogonal configuration, with a dihedral angle of 85.3°. The GaC_2_N_2_ and GaC_3_N rings exhibit perfect planarity, as evidenced by their minimal deviations from the least-squares plane (∆ = 0.021 and 0.129 Å). The Ga–N bond lengths (1.913(4) and 1.912(4) Å) to the α-diimine nitrogens are similar to the distance of Ga–N3 (1.929(4) Å) to the amido nitrogen atoms, which closely resembles observations of other related gallium amides [15]. A peak from difference maps that can be attributed to a N-bound proton, was refined to the N–H bond distance of 0.88 Å. Intramolecular C–H activation has been observed in Al and In amido derivatives [35,42], leading to the formation of a dianionic alkyl-amide ligand through C–H activation across a transient M–N_imide_ bond. The newly formed Ga–C bond length (1.995(5) Å) is comparable to the Ga–C bond lengths observed in aryl or alkyl derivatives of [NacNacGa] (range 1.9903(17)–2.0117(15) Å), which contain a single amide ligand [15].

DFT calculations on complex **6** (Figure S20) demonstrate a planar NC_3_ core, exhibiting bond orders of 1.21 for N3–C29, 1.68 for C29–C34, and 1.03 for C34–C37, thereby confirming the charge delocalization across the N–C=C–C unit. The WBIs of the Ga–N3 and Ga–C37 bonds (0.47 and 0.63) are slightly higher, compared to those observed for the Ga–N1 (0.43) and Ga–N2 (0.42) bonds. The Ga atom exhibits NPA charges of 1.65 e, while the ligand L displays negative charges of −1.41 e. The entire NC_3_ fragment shows negative charges of −1.23 e (Table S4, Supplementary Materials), primarily localized on the N3 (−1.02 e) and C37 (−0.98 e) atoms.

In addition, the reaction of **1** with 1.0 equivalent 2,6-diisopropylC_6_H_3_N_3_ led to the formation of the bis-amide complex [LGa(NH-2,6-*i*Pr_2_C_6_H_3_)_2_][Na(THF)_5_] (**7**, with low yield, 29% yield). Subsequently, altering the ratio of compound **1**: 2,6-diisopropylC_6_H_3_N_3_ to 1: 2 still led to the generation of compound **7** in the moderate yield (68% yield). We speculate that **6** and **7** are formed by essentially similar C–H activation processes, where the first is the result of intramolecular activation and the other is attributed to intermolecular hydrogen atom abstraction from the solvent (toluene) due to steric repulsion [18]. The structure of compound **7** is asymmetric due to the steric hindrance, as evidenced from the ^1^H NMR spectra. In particular, the *i*Pr methine protons exhibit two septets. The broad peak observed at 2.79 ppm is attributed to the presence of an NH functionality, while IR spectroscopy indicates the occurrence of a N–H stretch (*ν*_NH_ = 3364 cm^−1^).

**Figure 5:**
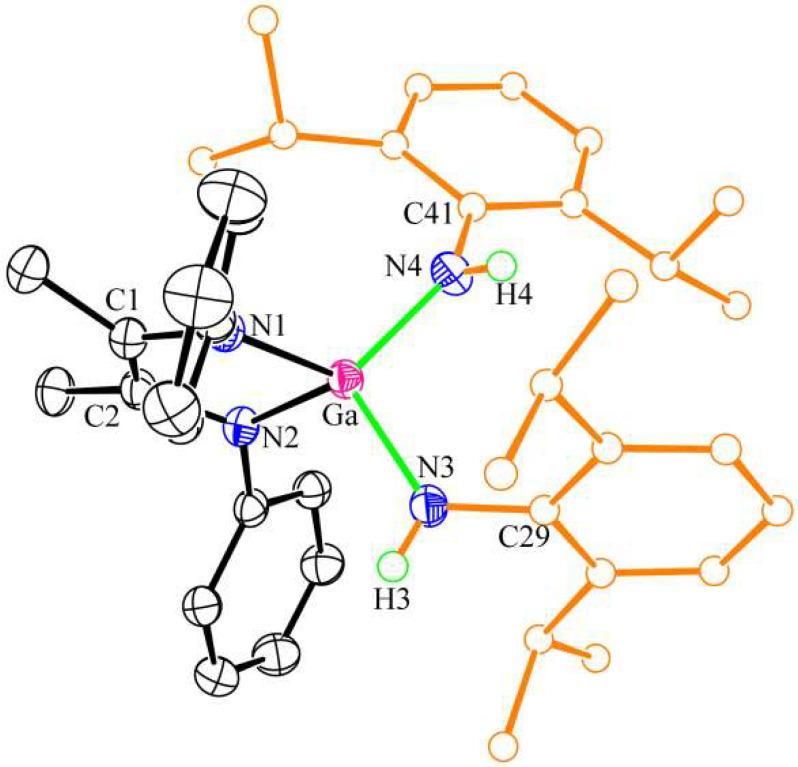
Molecular structure of **7**. Thermal ellipsoid plot (30%). H atoms except N3–H and N4–H, Na(THF)_5_ counterion, and the isopropyl groups are omitted for clarity. The green bonds represent newly formed chemical bonds. Selected bond lengths [Å] and angles [°]: Ga–N1 1.910(4), Ga–N2 1.915(4), N1–C1 1.417(4), N2–C2 1.424(5), C1–C2 1.353(5), Ga–N3 1.913(3), Ga–N4 1.908(4), N3–C29 1.397(5), N4–C41 1.401(4), N3–H3 0.8700, N4–H4 0.8700, N1-Ga-N2 88.05(19), N1-Ga-N3 118.07(14), N3-Ga-N2 107.3(2), N4-Ga-N1 116.2(2), N4-Ga-N2 123.07(15), N4-Ga-N3 104.3(2).

The structure of diamide complex **7** (Figure 5) reveals a gallium center coordinated to four nitrogen atoms in a distorted tetrahedral manner. The Ga–N bonds to α-diimine ligand (av. 1.911 Å) display similar lengths to the corresponding bonds in **1** (av. 1.918 Å). The α-diimine ring is folded along the N1···N2 axis, positioning Ga approximately 0.20 Å away from the averaged N_2_C_2_ plane, which makes the two bulk NH(Ar) ligands point away from the *i*Pr on the α-diimine ligand. The bond lengths of Ga–N(*i*Pr_2_C_6_H_3_) (1.913(3) and 1.908(4)Å) are similar to those observed for Ga–N(SiMe_3_)_2_ bonds (1.891(4), 1.884(1) and 1.872(2) Å in monoamide compounds [LGa(NHSiMe_3_)(benzyl)][Na(THF)_4_] [18], [(HC(MeCDippN)_2_)Ga(N_3_)N(SiMe_3_)_2_] [40], and [Cp*{N(SiMe_3_)_2_}Ga(μ-N_3_)]_2_ [47], probably due to larger steric hindrance of the amide moiety HN-(*i*Pr_2_C_6_H_3_) around the Ga–N bond. However, the lengths of these bonds exceed the distances reported for imides [Ar’GaNAr^#^] (1.701(3) Å, Ar’ = C_6_H_3_-2,6-(C_6_H_3_-2,6-*i*Pr_2_)_2_, Ar^#^ = C_6_H_3_-2,6-(C_6_H_2_-2,6-Me_2_-4-*t*Bu)_2_) [48] and [{HC(MeCDippN)_2_}GaN-2,6-Trip_2_C_6_H_3_] (1.742(3) Å, Trip = 2,4,6-*i*Pr_3_C_6_H_2_) [49] with a Ga=N double bond.

The NPA charges on the α-diimine ligand in model compound **7H** (−1.42 e) exhibit a similar range to those observed in **2H**–**7H** (−1.39~−1.42 e, Tables S3 and S4). The NPA charges on Ga (1.77 e) are somewhat higher than those observed in **2H**–**6H** (1.65–1.66 e). Accordingly, both the 2,6-diisopropylphenyl-amido groups exhibit negative partial charges of −0.68 e. The calculated Ga–N3 (1.943 Å) and Ga–N4 (1.935 Å) bond distances closely correspond to the experimental values (1.913(3) and 1.908(4) Å), exhibiting an average σ-bond order of 0.47, as revealed by NBO analysis.

## 3. Materials and Methods

### 3.1. General Procedures

All reactions and manipulations of air- and moisture-sensitive compounds were carried out under argon or nitrogen with standard Schlenk or dry box techniques. The solvents (ether, THF, toluene, and DME) were dried using appropriate methods and were distilled under argon prior to use. Tetrahydrofuran-*d*_8_ is dried over Na/K alloy. The α-diimine ligand L and [LGa][Na(THF)_3_] **1** L=[(2,6-*i*Pr_2_C_6_H_3_)NC(Me)]_2_ were prepared according to procedures outlined in the literature [50]. Sodium metal, Gallium chloride (GaCl_3_), Azobenzene, 4-Methoxyazobenzene, 4-Dimethylaminoazobenzene, and 15-Crown-5 were purchased from Alfa Aesar (Heysham, Britain). 3,3′-Dimethylazobenzene was purchased from Macklin (Shanghai, China). Azides were prepared from the corresponding amines via their diazonium salts [51]. The ^1^H and ^13^C NMR spectra were recorded on Bruker AVANCE III-400 MHz spectrometers (Rheinstetten, Germany). The IR spectra were recorded using a Thermo scientific Nicolet AVATAR 360 FT-IR (Thermo Scientific, Waltham, MA, USA). UV–visible spectra were recorded by an Agilent Tecgnologies Cary 100 UV–vis spectrophotometer (Santa Clara, CA, USA). Elemental analyses were carried out on Elementar VarioEL III instrument (Hanau, Germany). The X-ray diffraction data were collected with a Bruker METALJET D8 VENTURE (diffractometer with graphite-monochromated Ga Kα radiation) and Bruker APEX-IICCD (diffractometer with graphite-monochromated Mo Kα radiation) (Berlin, Germany). An empirical absorption correction using SADABS was applied for all data [52]. The structures were solved and refined to convergence on *F*^2^ for all independent reflections by the full-matrix least squares method using the SHELXL–2014 programs [53]. Some solvents, such as DME and THF, are disordered and display unusual thermal parameters, which were removed using the SQUEEZE routine implemented within the software program PLATON [54], and the resulting .fab file was processed with OLEX2 1.2 [55] using the ABIN instruction (see Supplementary Materials for X-ray crystal structure determination details). The structure optimization and NBO bonding analysis for the model compounds **2H−7H** were carried out at the DFT (B3LYP) level with the 6-31G* basis [56,57] sets using the Gaussian 09 program [58]. Bonding analyses were performed by means of natural bond orbital (NBO) analysis and natural population analysis (NPA). Wiberg bond indices (WBI) were evaluated with Weinhold’s natural bond orbital method [59,60,61] (see Supplementary Materials for DFT computations details). 

### 3.2. Synthesis of [LGa-κ^2^N,C-PhNN(H)(C_6_H_4_)][Na(DME)_3_] (***2***)

Azobenzene (0.183 g, 1 mmol) was added to the THF solution of compound **1** (1 mmol). The resultant black-green solution was stirred at room temperature for 1 day. All volatiles were removed in vacuum. The residue was dissolved in 5 mL of DME. After 5 days, the black-green crystals were grown by slow evaporation of the solution at room temperature (yield: 0.608 g, 0.64 mmol, 64%). ^1^H NMR (400 MHz, THF-*d*_8_, 298 K): *δ*/ppm: 0.54 (d, *J* = 6.8Hz, 6H, CH(C*H*_3_)_2_), 0.79 (d, *J* = 6.8 Hz, 6H, CH(C*H*_3_)_2_), 1.26 (m, 12H, CH(C*H*_3_)_2_) overlap with 6H, NCC*H*_3_), 3.26, 3.42 (DME), 3.98 (sept, *J* = 6.8 Hz, 2H, C*H*(CH_3_)_2_), 4.11 (sept, *J* = 6.9 Hz, 2H, C*H*(CH_3_)_2_), 5.52 (s, 1H, NH), 6.06 (t, *J* = 6.9 Hz, 1H, HNN*Ph*), 6.30 (t, *J* = 7.0 Hz 1H, NNHC_6_*H*_4_), 6.54 (m, 1H, Ar-H), 6.65–6.74 (m, 4H, Ar-H), 6.76–6.87 (m, 4H, Ar-H), 6.88–6.98 (m, 3H, Ar-H), 7.36 (d, *J* = 6.9 Hz, 1H, NNH-C_6_*H*_4_). ^13^C NMR (100.6 MHz, THF-*d*_8_, 298 K): δ/ppm: 15.7 (NC*C*H_3_), 24.6 (CH(*C*H_3_)_2_), 25.3 (THF-*d_8_*), 26.5, 26.9, 27.9 (CH(*C*H_3_)_2_), 28.8 (*C*H(CH_3_)_2_), 59.1, 72.7 (DME), 109.8 (N*C*CH_3_), 113.1, 117.5, 122.0, 122.6, 123.2, 123.8, 126.7, 138.0, 146.9, 147.5, 149.1, 152.8, 156.5, 157.2 (Ar-C). IR (Nujol, KBr, ν/cm^−1^): 3338 w, 3073 m, 2971 s, 2851 s, 1596 m, 1494 s, 1417 s, 1323 m, 1246 s, 1177 w, 1049 m, 793 m. Elemental analysis calcd. for C_52_H_80_GaN_4_NaO_6_ (949.91): C 65.75; H 8.49; N 5.90%; found: C 65.36, H 8.73, N 5.59%.

### 3.3. Synthesis of [LGa-κ^2^N,C-PhNN(H)(p-OCH_3_-C_6_H_3_)][Na(DME)_3_] (***3***)

Complex **3** was prepared by a similar procedure to that of **2**. 4-Methoxyazobenzene (0.213 g, 1 mmol) was added to the THF solution of compound **1** (1 mmol). The resultant black-green solution was stirred at room temperature for 1 day. All volatiles were removed in vacuum. The residue was dissolved in 5 mL of DME. After 4 days, the black-green crystals were grown by slow evaporation of the solution at room temperature (yield: 0.667 g, 0.68 mmol, 68%). ^1^H NMR (400 MHz, THF-*d_8_*, 298 K): *δ*/ppm: 0.53 (d, *J* = 6.7 Hz, 6H, CH(C*H*_3_)_2_), 0.80 (d, *J* = 6.7 Hz, 6H, CH(C*H*_3_)_2_), 1.27–1.31 (m, 12H, CH(C*H*_3_)_2_ overlap with 6H, NCC*H*_3_), 3.26, 3.42 (DME), 3.53 (s, 3H, OC*H*_3_), 3.99 (sept, *J* = 6.7 Hz, 2H, C*H*(CH_3_)_2_), 4.10 (sept, *J* = 6.6 Hz, 2H, C*H*(CH_3_)_2_), 5.18 (s, 1H, NH), 6.07 (t, *J* = 6.7 Hz, 1H, NH-N-C_6_*H*_3_), 6.34 (dd, *J* = 8.4 Hz, 1H, Ar-H), 6.46 (B, 1H, Ar-H), 6.50 (d, *J* = 8.5 Hz, 2H, Ar-H), 6.62 (d, *J* = 8.4 Hz, 1H, Ar-H), 6.69 (m, 4H, Ar-H), 6.86 (m, 3H, Ar-H), 6.99 (s, 1H, NH-C_6_H_3_). ^13^C NMR (100.6 MHz, THF-*d_8_*, 298 K): *δ*/ppm: 14.8 (NC*C*H_3_), 25.3 (THF-*d_8_*), 24.6, 26.7 26.9 (CH(*C*H_3_)_2_), 28.1 (*C*H(CH_3_)_2_)), 55.3 (O*C*H_3_), 59.0, 72.5 (DME), 67.4 (THF-*d_8_*), 106.9 (N*C*CH_3_), 109.9, 114.2, 115.4, 116.6, 117.5, 120.4, 122.1, 122.8, 126.6, 127.5, 128.6, 148.0, 151.7 (Ar-C), 153.1 (Ar-*C*-OCH_3_). IR (Nujol, KBr, ν/cm^−1^): 3252 w, 3056 vs, 2919 vs, 2715 w, 2569 w, 2407 w, 2347 w, 2228 s, 2065 s, 1886 w, 1766 w, 1596 vs, 1494 vs, 1442 vs, 1067 vs, 1032 vs, 827 vs, 785 vs, 716 vs, 673 s. Elemental analysis C_92_H_124_Ga_2_N_12_Na_2_O_2_ (1615.44): calc. C 68.40, H 7.74, N 10.40%; found: C 68.31, H 7.92, N 10.21%. Elemental analysis calcd. for C_53_H_82_GaN_4_NaO_7_ (979.93): C 64.96; H 8.43; N 5.72%; found: C 64.49, H 8.65, N 5.34%.

### 3.4. Synthesis of [LGa-κ^2^N,C-PhNN(H)(p-N(CH_3_)_2_-C_6_H_3_)][Na(THF)_3_] (***4***)

4-Dimethylaminoazobenzene (0.226 g, 1 mmol) was added to the THF solution of compound **1** (1 mmol). The resultant black-green solution was stirred at room temperature for 1 day and concentrated to about 5 mL under reduced pressure. A few drops of DME were added. After 7 days, the black-green crystals were grown by slow evaporation of the solution at room temperature (yield: 0.658 g, 0.70 mmol, 70%). ^1^H NMR (400 MHz, THF-*d_8_*, 298 K): *δ*/ppm: 0.49 (d, *J* = 6.6 Hz, 6H, CH(C*H*_3_)_2_), 0.75 (d, *J* = 6.8 Hz, 6H, CH(C*H*_3_)_2_), 0.94 (m, 6H, CH(C*H*_3_)_2_), 1.15 (s, 3H, NCC*H*_3_), 1.17 (s, 3H, NCC*H*_3_), 1.24 (m, 12H, CH(C*H*_3_)_2_)), 1.58 (s, 6H, NCC*H*_3_), 1.69 (Na-THF), 2.02 (toluene), 3.23, 3.40 (DME), 3.47 (Na-THF), 3.95 (sept, *J* = 6.6 Hz, 2H, C*H*(CH_3_)_2_), 4.05 (sept, *J* = 6.8 Hz, 2H, C*H*(CH_3_)_2_), 5.12 (s, 1H, NH), 6.05 (t, 1H, NHNC_6_*H*_5_), 6.32 (m, 1H, Ar-H), 6.47 (d, 2H, Ar-H), 6.67 (m, toluene), 6.79 (m, 5H, Ar-H), 6.93 (m, 3H, Ar-H), 7.11 (m, 3H, Ar-H). ^13^C NMR (100.6 MHz, THF-*d_8_*, 298 K): δ/ppm: 16.5 (NC*C*H_3_), 22.8, 23.3 (CH(*C*H_3_)_2_), 25.3 (THF-*d_8_*), 26.8, 71.5(THF), 29.3 (*C*H(CH_3_)_2_), 46.2 (N(*C*H_3_)_2_), 58.9, 72.7 (DME), 67.4 (THF-*d_8_*), 122.1 (N*C*CH_3_), 122.7 (Ga-C), 123.6, 124.5, 135.5, 147.1, 168.9 (Ar-C). IR (Nujol, KBr, ν/cm^−1^): 3380 w, 3056 m, 2843 vs, 1596 s, 1425 vs, 1357 s, 1237 vs, 1160 m, 1092 vs, 939 m, 785 m. Elemental analysis calcd. for C_54_H_79_GaN_5_NaO_3_ (938.93): C 69.07; H 8.48; N 7.46%; found: C 68.87, H 8.69, N 7.03%.

### 3.5. Synthesis of [LGa-κ^2^N,C-(m-CH_3_-C_6_H_4_)NN(H)(m-CH_3_-C_6_H_3_)][Na(15-C-5)_2_] (***5***)

In this step, 15-Crown-5 (300 µL, 1 mmol) was added to the THF solution of compound **1** (1 mmol) and stirred at room temperature for 1 day; then, 3,3′-dimethylazobenzene (0.211 g, 1mmol) was added. The resultant green solution was stirred at room temperature for 1 day and concentrated to about 5 mL under reduced pressure. After 5 days, the green crystals were grown by slow evaporation of the solution at room temperature (yield: 0.827 g, 0.72 mmol, 72%). ^1^H NMR (400 MHz, THF-*d*_8_, 298 K): *δ*/ppm: 0.54 (d, *J* = 6.7 Hz, 6H, CH(C*H*_3_)_2_), 0.80 (d, *J* = 6.7 Hz, 6H, CH(C*H*_3_)_2_), 1.27 (d, *J* = 6.8 Hz, 12H, CH(C*H*_3_)_2_ overlap with s, 6H, NCC*H*_3_), 2.07 (s, 3H, C_6_H_3_-C*H*_3_), 2.17 (s, 2H, C_6_H_3_-C*H*_3_), 2.22 (s, 1H, C_6_H_3_-C*H*_3_), 3.48 (15-C-5-*H*), 3.98 (b, 2H, C*H*(CH_3_)_2_), 4.10 (b, 2H, C*H*(CH_3_)_2_), 5.51 (s, 1H, NH), 5.94 (d, *J* = 6.8 Hz, 1H, C_6_*H*_3_-CH_3_), 6.20 (d, 2H, C_6_*H*_3_-CH_3_), 6.39 (s, 1H, C_6_*H*_3_-CH_3_), 6.70 (m, 6H, Ar-H), 6.82 (m, 2H, C_6_*H*_3_-CH_3_), 7.25 (d, *J* = 6.8 Hz, 1H, C_6_*H*_3_-CH_3_). ^13^C NMR (100.6 MHz, THF-*d*_8_, 298 K): *δ*/ppm: 21.9 (NC*C*H_3_), 24.7, 26.5 (Ar-*C*H_3_), 27.1 (CH(*C*H_3_)_2_), 28.0 (*C*H(CH_3_)_2_), 70.5 (15-C-5-C), 103.8 (N*C*CH_3_), 107.5, 111.1, 113.8, 117.6, 119.1, 122.2, 122.2, 127.4, 128.5, 135.7, 138.0, 152.7, 157.8 (Ar-C). IR (Nujol, KBr, ν/cm^−1^): 3235 w, 3030 s, 2902 vs, 1971 w, 1613 s, 1425 vs, 1331 s, 1237 s, 1083 vs, 930 s, 768 m. Elemental analysis calcd. for C_62_H_94_GaN_4_NaO_10_ (1148.12): C 64.86; H 8.25; N 4.88%; found: C 64.74, H 8.30, N 4.65%.

### 3.6. Synthesis of [LGa(NH-2-(CH_2_)-4,6-Me_2_C_6_H_2_)][Na(15-C-5)_2_] (***6***)

An amount of 300 µL of 15-Crown-5 was added to the toluene solution of compound **1** (1 mmol) and stirred at room temperature for 1 day; then, 2,4,6-trimethylN_3_ (0.223 g, 1 mmol) was added. The solution gradually changes from orange-red to brown-red; this is accompanied by the release of nitrogen. The reaction solution was stirred at room temperature for 1 day and concentrated to about 5 mL under reduced pressure. A few drops of THF were added. The yellow crystal of **6** (yield: 0.800 g, 0.75 mmol, 75%) was obtained in 8 days through the process of slow evaporation at room temperature. ^1^H NMR (400 MHz, THF-*d_8_*, 298 K): *δ*/ppm: 0.99 (s, 2H, Ga-C*H*_2_ overlap with d, *J* = 6.8 Hz, 6H, CH(C*H*_3_)_2_), 1.13 (dd, *J* = 6.7 Hz, 12H, C(C*H*)_3_)_2_), 1.18 (d, *J* = 6.8 Hz, 6H, C(C*H*)_3_)_2_), 1.51 (s, 6H, NCC*H*_3_), 1.92 (s, 6H, C_6_H_2_-(C*H*_3_)_2_), 3.21 (s, 1H, NH), 3.47 (15-C-5-*H*), 3.87 (sept, *J* = 6.8 Hz, 2H, C*H*(CH_3_)_2_), 4.19 (sept, *J* = 6.9 Hz, 2H, C*H*(CH_3_)_2_), 6.16 (s, 1H, NH-C_6_*H*_2_), 6.34 (s, 1H, NH-C_6_*H*_2_), 6.74 (t, *J* = 7.5 Hz, 2H, Ar-H) and 6.86 (d,d, *J* = 7.4 Hz, 4H, Ar-H). ^13^C NMR (100.6 MHz, THF-*d_8_*, 298 K): *δ*/ppm: 10.3 (Ga-*C*H_2_), 15.4 (NC*C*H_3_), 19.3 (N-phenyl-p-*C*H_3_), 21.2 (N-phenyl-o-*C*H_3_), 21.5, 24.7, 26.1, 26.2, 27.5(CH(*C*H_3_)_2_), 27.7 (*C*H(CH_3_)_2_), 70.4 (15-C-5-C), 116.0, 116.5 (N*C*CH_3_), 117.6, 121.8, 122.4, 122.6, 126.0, 126.6, 128.9, 128.9, 129.7, 138.4, 148.4, 148.7, 149.6, 155.2 (Ar-C). IR (Nujol, KBr, ν/cm^−1^): 3499 m, 3176 w, 2962 vs, 2159 w, 1920 m, 1630 vs, 1433 vs, 1229 vs, 1101 vs, 913 vs, 759 vs. Elemental analysis calcd. for C_57_H_91_GaN_3_NaO_10_ (1071.03): C 63.92, H 8.56 N 3.92%; found: C 63.99, H 8.65, N 3.82%.

### 3.7. Synthesis of [LGa(NH-2,6-iPr_2_C_6_H_3_)_2_][Na(THF)_5_] (***7***)

2,6-*i*Pr_2_C_6_H_3_N_3_ (0.408 g, 2 mmol) was added to the toluene solution of compound **1** (1 mmol). The solution gradually changes from orange-red to brown-red; this process is accompanied by the release of nitrogen. The reaction solution was stirred at room temperature for 1 day and concentrated to about 5 mL under reduced pressure. A few drops of THF were added. The yellow crystal of **7** (yield: 0.823 g, 0.68 mmol, 68%) was obtained in 8 days through the process of slow evaporation at room temperature. ^1^H NMR (400MHz, THF-d*_8_*, 298 K): *δ*/ppm: 0.73 (d, *J* = 6.4 Hz, 24H, NH-CH(C*H*_3_)_2_), 0.94 (d, *J* = 6.6 Hz, 12H, CH(C*H*_3_)_2_), 1.01 (d, *J* = 6.6 Hz, 12H, CH(C*H*_3_)_2_), 1.61 (s, 6H, NCC*H*_3_), 1.74 (m, Na-THF overlap with THF-d_8_), 2.69 (br, 4H, C*H*(CH_3_)_2_), 2.79 (s, 2H, NH), 3.24, 3.40 (DME), 3.58 (m, Na-THF overlap with THF-d_8_), 4.14 (sept, *J* = 6.7 Hz, 4H, C*H*(CH_3_)_2_), 6.23 (m, 2H, Ar-H), 6.60 (m, 2H, Ar-H), 6.76 (m, 2H, Ar-H), 6.91 (m, 2H, Ar-H). ^13^C NMR (100.6 MHz,THF-*d_8_*, 298 K): *δ*/ppm: 16.2 (NC*C*H_3_), 22.9 (Toulene), 26.4 (THF), 27.5 (CH(*C*H_3_)_2_), 27.8 (*C*H(CH_3_)_2_), 58.9 (DME), 68.2 (THF), 72.7 (DME), 114.7 (N*C*CH_3_), 121.7, 122.9, 122.3, 123.5, 148.3, 150.6 (Ar-C). IR (Nujol, KBr, ν/cm^−1^): 3364 m, 2902 vs, 2219 m, 1579 m, 1442 vs, 1348 vs, 1254 vs, 1083 m, 990 s, 742 s. Elemental analysis calcd. for C_72_H_116_GaN_4_NaO_5_ (1210.39): C 71.44; H 9.66; N 4.63%; found: C 71.42, H 9.54, N 4.62%.

## 4. Conclusions

The anionic gallylene species [LGa:]·[Na(THF)_3_] (**1**) demonstrates the ability to selectively activate sp^2^ C−H bonds of azobenzene derivatives. The presence of electron-donating groups (–OMe, –N(Me)_2_, –Me) attached to azobenzene facilitates the formation of anionic complexes **2**−**5**. This process involves the activation of an ortho-C–H bond within a phenyl group, followed by migration of the hydrogen atom to a nitrogen atom. We further investigated the reactions of azides RN_3_ (R = 2,4,6-trimethylphenyl, 2, 6-diisopropylphenyl) with gallylene **1**, resulting in the formation of a dianionic alkyl-amide (in **6**) or bis-amide (in **7**) ligand through intramolecular or intermolecular hydrogen atom abstraction across a transient Ga=N imide bond.

## Figures and Tables

**Table 1:** Selected bond lengths (Å) and angles (°) for complexes **2**−**5**.

Compounds	2	3	4	5
Bond length [Å]				
Ga-N1	1.911(2)	1.887(8)	1.888(2)	1.901(9)
Ga–N2	1.897(2)	1.872(8)	1.894(2)	1.883(9)
C1–N1	1.418(4)	1.415(1)	1.428(3)	1.395(14)
C2–N2	1.418(4)	1.453(1)	1.421(3)	1.435(14)
C1–C2	1.355(4)	1.339(2)	1.354(4)	1.334(10)
Ga–N3	1.965(3)	2.026(1)	2.017(2)	1.963(6)
Ga–C36	1.967(3)	1.916(1)	1.959(2)	1.952(7)
N3–N4	1.426(4)	1.374(1)	1.354(3)	1.417(8)
N3–C29	1.366(4)	1.363(2)	1.388(3)	1.368(9)
N4–C35	1.404(4)	1.355(1)	1.387(3)	1.408(9)
C35–C36	1.404(4)	1.442(2)	1.416(4)	1.388(9)
N4–H	0.8800	0.8800	0.8800	0.8700
Bond angles [°]				
N1-Ga-N2	87.1(1)	88.7(4)	88.2(9)	87.4(2)
N2-Ga-N3	114.5(1)	114.5(4)	116.50(9)	114.4(5)
N2-Ga-C36	129.1(1)	126.5(4)	125.2(1)	128.2(5)
N1-Ga-N3	113.6(1)	115.4(4)	112.6(9)	114.1(5)
N3-Ga-C36	85.7(1)	83.5(5)	83.9(10)	85.4(2)
N1-Ga-C36	128.9(1)	130.3(5)	132.6(10)	129.4(5)

## Data Availability

Data are contained within the article and in the Supplementary Materials.

## Supplementary Materials

The following supporting information can be downloaded at: https://www.mdpi.com/article/10.3390/molecules29215021/s1, X-ray crystal structure determination details, crystal data, and DFT computations. Figures S1–S12: ^1^H NMR and ^13^C NMR spectrums of **2**−**7**. Figures S13–S18: UV–vis spectra of **2**−**7**. Figure S19: Molecular structure of **3**. Figure S20: Optimized structures of **2H**−**7H** labelled with selected bond orders. Table S1 and S2: Crystallographic data and refinement details for compounds **2**−**7**. Tables S3 and S4: Natural charges (e) of the model compounds **2H**–**7H**. DFT computations.

## References

[1] Sinha S.K., Guin S., Maiti S., Biswas J.P., Porey S., Maiti D. (2022). Toolbox for Distal C–H Bond Functionalizations in Organic Molecules. Chem. Rev..

[2] Ackermann L. (2011). Carboxylate-Assisted Transition-Metal-Catalyzed C–H Bond Functionalizations: Mechanism and Scope. Chem. Rev..

[3] Shang R., Ilies L., Nakamura E. (2017). Iron-Catalyzed C–H Bond Activation. Chem. Rev..

[4] Yang Y., Wu Y., Bin Z., Zhang C., Tan G., You J. (2024). Discovery of Organic Optoelectronic Materials Powered by Oxidative Ar–H/Ar–H Coupling. J. Am. Chem. Soc..

[5] Engle K.M., Mei T.-S., Wasa M., Yu J.-Q. (2012). Weak Coordination as a Powerful Means for Developing Broadly Useful C–H Functionalization Reactions. Acc. Chem. Res..

[6] Rungthanaphatsophon P., Barnes C.L., Kelley S.P., Walensky J.R. (2018). Four-electron reduction chemistry using a uranium(iii) phosphido complex. Dalton Trans..

[7] Fischer R.C., Power P.P. (2010). π-Bonding and the Lone Pair Effect in Multiple Bonds Involving Heavier Main Group Elements: Developments in the New Millennium. Chem. Rev..

[8] Judge N.R., Logallo A., Hevia E. (2023). Main group metal-mediated strategies for C–H and C–F bond activation and functionalisation of fluoroarenes. Chem. Sci..

[9] Hicks J., Vasko P., Goicoechea J.M., Aldridge S. (2018). Synthesis, structure and reaction chemistry of a nucleophilic aluminyl anion. Nature.

[10] Hooper T.N., Garçon M., White A.J.P., Crimmin M.R. (2018). Room temperature catalytic carbon–hydrogen bond alumination of unactivated arenes: Mechanism and selectivity. Chem. Sci..

[11] Brand S., Elsen H., Langer J., Grams S., Harder S. (2019). Calcium-Catalyzed Arene C–H Bond Activation by Low-Valent AlI. Angew. Chem. Int. Ed..

[12] Chu T., Korobkov I., Nikonov G.I. (2014). Oxidative Addition of σ Bonds to an Al(I) Center. J. Am. Chem. Soc..

[13] Chen W., Liu L., Zhao Y., Xue Y., Xu W., Li N., Wu B., Yang X.-J. (2021). Organometallo-macrocycle assembled through dialumane-mediated C–H activation of pyridines. Chem. Commun..

[14] Kassymbek A., Vyboishchikov S.F., Gabidullin B.M., Spasyuk D., Pilkington M., Nikonov G.I. (2019). Sequential Oxidation and C–H Bond Activation at a Gallium(I) Center. Angew. Chem. Int. Ed..

[15] Kassymbek A., Spasyuk D., Dmitrienko A., Pilkington M., Nikonov G.I. (2022). Facile C–H bond activation on a transient gallium imide. Chem. Commun..

[16] Puthiyaveetil S.S., Kassymbek A., Dmitrienko A., Pilkington M., Nikonov G.I. (2023). 1,3-C–H bond activation on a transient gallium(i)/isocyanate adduct. Dalton Trans..

[17] Sharma M.K., Wölper C., Haberhauer G., Schulz S. (2021). Multi-Talented Gallaphosphene for Ga–P–Ga Heteroallyl Cation Generation, CO_2_ Storage, and C(sp3)–H Bond Activation. Angew. Chem. Int. Ed..

[18] Zhang Y., Dodonov V.A., Chen W., Zhang S., Roesky P.W., Zhao Y., Fedushkin I.L., Yang X.-J. (2023). Reactions of Low-Valent Gallium Species with Organic Azides: Formation of Imido-, Azoimido-, and Tetrazene Complexes. Inorg. Chem..

[19] Rudenko A.E., Clayman N.E., Walker K.L., Maclaren J.K., Zimmerman P.M., Waymouth R.M. (2018). Ligand-Induced Reductive Elimination of Ethane from Azopyridine Palladium Dimethyl Complexes. J. Am. Chem. Soc..

[20] Mehrparvar S., Scheller Z.N., Wölper C., Haberhauer G. (2021). Design of Azobenzene beyond Simple on–off Behavior. J. Am. Chem. Soc..

[21] Nguyen T.H.L., Gigant N., Joseph D. (2018). Advances in Direct Metal-Catalyzed Functionalization of Azobenzenes. ACS Catal..

[22] Tylkowski B., Trojanowska A., Marturano V., Nowak M., Marciniak L., Giamberini M., Ambrogi V., Cerruti P. (2017). Power of light-Functional complexes based on azobenzene molecules. Coord. Chem. Rev..

[23] Kaim W. (2001). Complexes with 2,2’-azobispyridine and related ‘S-frame’ bridging ligands containing the azo function. Coord. Chem. Rev..

[24] Henwood A.F., Hu Y., Sajjad M.T., Thalluri K.V.V., Ghosh S.S., Cordes D.B., Slawin A.M.Z., Samuel I.D.W., Robertson N., Zysman-Colman E. (2015). Unprecedented Strong Panchromic Absorption from Proton-Switchable Iridium(III) Azoimidazolate Complexes. Chem. Eur. J..

[25] Baker R.J., Jones C., Mills D.P., Murphy D.M., Hey-Hawkins E., Wolf R. (2006). The reactivity of gallium-(i), -(ii) and -(iii) heterocycles towards Group 15 substrates: Attempts to prepare gallium–terminal pnictinidene complexes. Dalton Trans..

[26] Azhakar R., Roesky H.W., Wolf H., Stalke D. (2012). Reactivity of Stable Heteroleptic Silylene PhC(NtBu)_2_SiNPh_2_ toward Diazobenzene and N-Benzylidineaniline. Organometallics.

[27] Allen F.H., Kennard O., Watson D.G., Brammer L., Orpen A.G., Taylor R. (1987). Tables of bond lengths determined by X-ray and neutron diffraction. Part 1. Bond lengths in organic compounds. J. Chem. Soc. Perkin Trans. II.

[28] Khanum R., Ra S.A., Rangaswamy H.R., Santhosh Kumar S.R., Prashanth Kumar A.G., Jagadisha A.S. (2023). Recent review on Synthesis, spectral Studies, versatile applications of azo dyes and its metal complexes. Results Chem..

[29] Su Y., Kinjo R. (2019). Small molecule activation by boron-containing heterocycles. Chem. Soc. Rev..

[30] Chu T., Nikonov G.I. (2018). Oxidative Addition and Reductive Elimination at Main-Group Element Centers. Chem. Rev..

[31] Yao S., Saddington A., Xiong Y., Driess M. (2023). Chelating Bis-silylenes as Powerful Ligands to Enable Unusual Low-Valent Main-Group Element Functions. Acc. Chem. Res..

[32] Dodonov V.A., Kushnerova O.A., Baranov E.V., Fedushkin I.L. (2024). Reduction and Cycloaddition of Heteroalkenes at Ga(I) Bisamide Center. Reactions.

[33] Fedushkin I.L., Dodonov V.A., Skatova A.A., Sokolov V.G., Piskunov A.V., Fukin G.K. (2018). Redox-Active Ligand-Assisted Two-Electron Oxidative Addition to Gallium(II). Chem. Eur. J..

[34] Zhu L., Kinjo R. (2023). Reactions of main group compounds with azides forming organic nitrogen-containing species. Chem. Soc. Rev..

[35] Anker M.D., Lein M., Coles M.P. (2019). Reduction of organic azides by indyl-anions. Isolation and reactivity studies of indium–nitrogen multiple bonds. Chem. Sci..

[36] Zhu H., Yang Z., Magull J., Roesky H.W., Schmidt H.-G., Noltemeyer M. (2005). Syntheses and Structural Characterization of a LAl(N_3_)N[μ-Si(N_3_)(tBu)]_2_NAl(N_3_)L and a Monomeric Aluminum Hydride Amide LAlH(NHAr) (L = HC[(CMe)(NAr)]_2_, Ar = 2,6-*i*Pr_2_C_6_H_3_). Organometallics.

[37] Cui C., Roesky H.W., Schmidt H.-G., Noltemeyer M. (2000). [HC{(CMe)(NAr)}_2_]Al[(NSiMe_3_)_2_N_2_] (Ar=2,6-*i*Pr_2_C_6_H_3_): The First Five-Membered AlN4 Ring System. Angew. Chem. Int. Ed..

[38] Queen J.D., Irvankoski S., Fettinger J.C., Tuononen H.M., Power P.P. (2021). A Monomeric Aluminum Imide (Iminoalane) with Al–N Triple-Bonding: Bonding Analysis and Dispersion Energy Stabilization. J. Am. Chem. Soc..

[39] Heilmann A., Hicks J., Vasko P., Goicoechea J.M., Aldridge S. (2020). Carbon Monoxide Activation by a Molecular Aluminium Imide: C–O Bond Cleavage and C–C Bond Formation. Angew. Chem. Int. Ed..

[40] Hardman N.J., Power P.P. (2001). Unique structural isomerism involving tetrazole and amide/azide derivatives of gallium. Chem. Commun..

[41] Yu Q., Zhang L., He Y., Pan J., Li H., Bian G.-q., Chen X., Tan G. (2021). A stable tetrazagallole and its radical anion dimer. Chem. Commun..

[42] Zhu H., Chai J., Chandrasekhar V., Roesky H.W., Magull J., Vidovic D., Schmidt H.-G., Noltemeyer M., Power P.P., Merrill W.A. (2004). Two Types of Intramolecular Addition of an Al–N Multiple-Bonded Monomer LAlNAr’ Arising from the Reaction of LAl with N_3_Ar’ (L = HC[(CMe)(NAr)]_2_, Ar’ = 2,6-Ar_2_C_6_H_3_, Ar = 2,6-*i*Pr_2_C_6_H_3_). J. Am. Chem. Soc..

[43] Zhu H., Chai J., Jancik V., Roesky H.W., Merrill W.A., Power P.P. (2005). The Selective Preparation of an Aluminum Oxide and Its Isomeric C–H-Activated Hydroxide. J. Am. Chem. Soc..

[44] Baek Y., Betley T.A. (2019). Catalytic C–H Amination Mediated by Dipyrrin Cobalt Imidos. J. Am. Chem. Soc..

[45] Stroek W., Keilwerth M., Pividori D.M., Meyer K., Albrecht M. (2021). An Iron–Mesoionic Carbene Complex for Catalytic Intramolecular C–H Amination Utilizing Organic Azides. J. Am. Chem. Soc..

[46] Lu H., Lang K., Jiang H., Wojtas L., Zhang X.P., Lu H., Lang K., Jiang H., Wojtas L., Zhang X.P. (2016). Intramolecular 1, 5-C (sp^3^)–H radical amination via Co (II)-based metalloradical catalysis for five-membered cyclic sulfamides. Chem. Sci..

[47] Jutzi P., Neumann B., Reumann G., Stammler H.-G. (1999). Novel Ga_2_N_2_ Ring Systems by Reaction of Pentamethylcyclopentadienylgallium with Organic Azides. Organometallics.

[48] Wright R.J., Brynda M., Fettinger J.C., Betzer A.R., Power P.P. (2006). Quasi-Isomeric Gallium Amides and Imides GaNR_2_ and RGaNR (R = Organic Group):  Reactions of the Digallene, Ar’GaGaAr’ (Ar’ = C_6_H3-2,6-(C_6_H_3_-2,6-*i*Pr_2_)_2_) with Unsaturated Nitrogen Compounds. J. Am. Chem. Soc..

[49] Hardman N.J., Cui C., Roesky H.W., Fink W.H., Power P.P. (2001). Stable, Monomeric Imides of Aluminum and Gallium: Synthesis and Characterization of [{HC(MeCDippN)_2_}MN-2,6-Trip_2_C_6_H_3_] (M=Al or Ga; Dipp=2,6-*i*Pr_2_C_6_H_3_; Trip=2,4,6-*i*Pr_3_C_6_H_2_). Angew. Chem. Int. Ed..

[50] Liu Y., Li S., Yang X.-J., Li Q.-S., Xie Y., Schaefer H.F., Wu B. (2011). Alkali metal compounds of a gallium(I) carbene analogue {:Ga[N(Ar)C(Me)]_2_} (Ar = 2,6-*i*Pr_2_C_6_H_3_). J. Organomet. Chem..

[51] Kitamura M., Yano M., Tashiro N., Miyagawa S., Sando M., Okauchi T. (2011). Direct Synthesis of Organic Azides from Primary Amines with 2-Azido-1,3-dimethylimidazolinium Hexafluorophosphate. Eur. J. Org. Chem..

[52] Sheldrick G.M. (2016). SADABS v.2016/2, Bruker/Siemens Area Detector Absorption Correction Program.

[53] Sheldrick G. (2015). Crystal structure refinement with SHELXL. Acta Crystallogr. Sect. C.

[54] Spek A.L. (2015). PLATON SQUEEZE: A tool for the calculation of the disordered solvent contribution to the calculated structure factors. Acta Cryst..

[55] Dolomanov O.V., Bourhis L.J., Gildea R.J., Howard J.A.K., Puschmann H. (2009). OLEX2: A complete structure solution, refinement and analysis program. J. Appl. Crystallogr..

[56] Becke A.D. (1993). Density-functional thermochemistry. III. The role of exact exchange. J. Chem. Phys..

[57] Lee C., Yang W., Parr R.G. (1988). Development of the Colle-Salvetti correlation-energy formula into a functional of the electron density. Phys. Rev. B.

[58] Frisch M.J., Trucks G.W., Schlegel H.B., Scuseria G.E., Robb M.A., Cheeseman J.R., Scalmani G., Barone V., Mennucci B., Petersson G. (2009). Gaussian 09, Revision C.1.

[59] Sizova O.V., Skripnikov L.V., Sokolov A.Y. (2008). Symmetry decomposition of quantum chemical bond orders. J. Mol. Struct. THEOCHEM.

[60] Reed A.E., Curtiss L.A., Weinhold F. (1988). Intermolecular interactions from a natural bond orbital, donor-acceptor viewpoint. Chem. Rev..

[61] Wiberg K.B. (1968). Application of the pople-santry-segal CNDO method to the cyclopropylcarbinyl and cyclobutyl cation and to bicyclobutane. Tetrahedron.

