# Supplementary material for: Reactions of an Anionic Gallylene with Azobenzene or Azide Compounds Through C(sp2)–H and C(sp3)–H Activation
Source: Molecules. 2024 Oct 24;29(21):5021. doi: 10.3390/molecules29215021 (PMC11547653; doi:10.3390/molecules29215021)
Supplement: Supplementary file 1 [file molecules-29-05021-s001.zip › molecules-3252656-supplementary.pdf]

Supplementary Materials

Reactions of an Anionic Gallylene with Azobenzene or Azide Compounds through C(sp^2)−H and C(sp^3)−H activation

Jinfeng Sun,[1,†] Fangfeng Chen,[1,†] Juan Liu[1,†] Yihu Zhang,[1] Dongyu He,[1]

Vladimir A. Dodonov,[1,2] Yanxia Zhao[1,]*

[1] Key Laboratory of Synthetic and Natural Functional Molecule of the Ministry of Education, College of Chemistry and Materials Science, Northwest University, Xi'an 710069, China

[2] Grigory Alekseevich Razuvaev Institute of Organometallic Chemistry of Russian Academy of Sciences (IOMC RAS), Tropinina 49, Nizhny Novgorod 603950, Russian Federation

[†] These authors contributed equally to this work.

* Correspondence: zhaoyx@nwu.edu.cn (Y. Z.)

Table of Contents

S1. NMR and UV−vis Spectra

S2. X-ray Crystallographic Analysis

S3. Theoretical Calculations

S4. References

S1. NMR and UV−vis Spectra

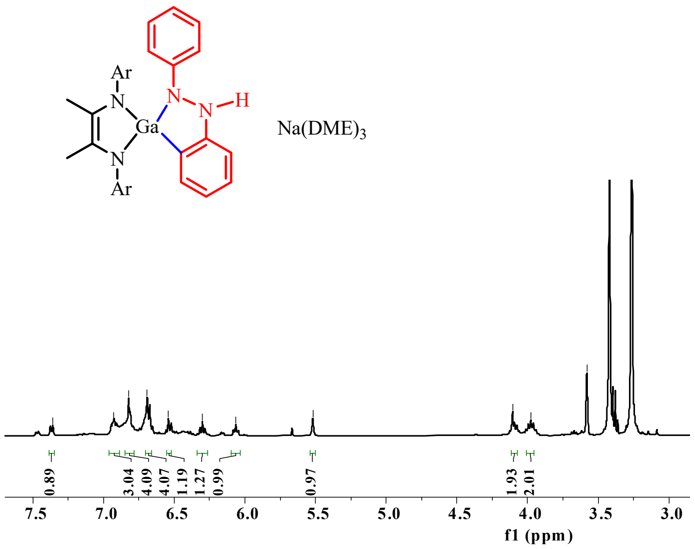

Figure S1. ^{1}H NMR spectrum of **2** (THF-d_8, 298 K).

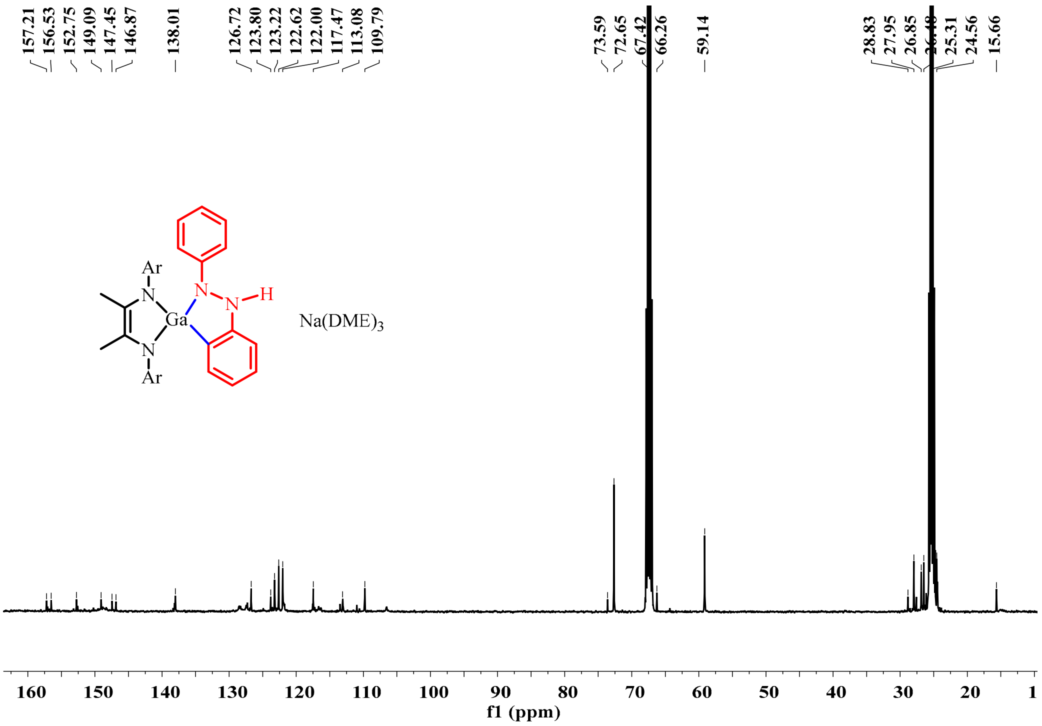

Figure S2. ^{13}C NMR spectrum of **2** (THF-d_8, 298 K).

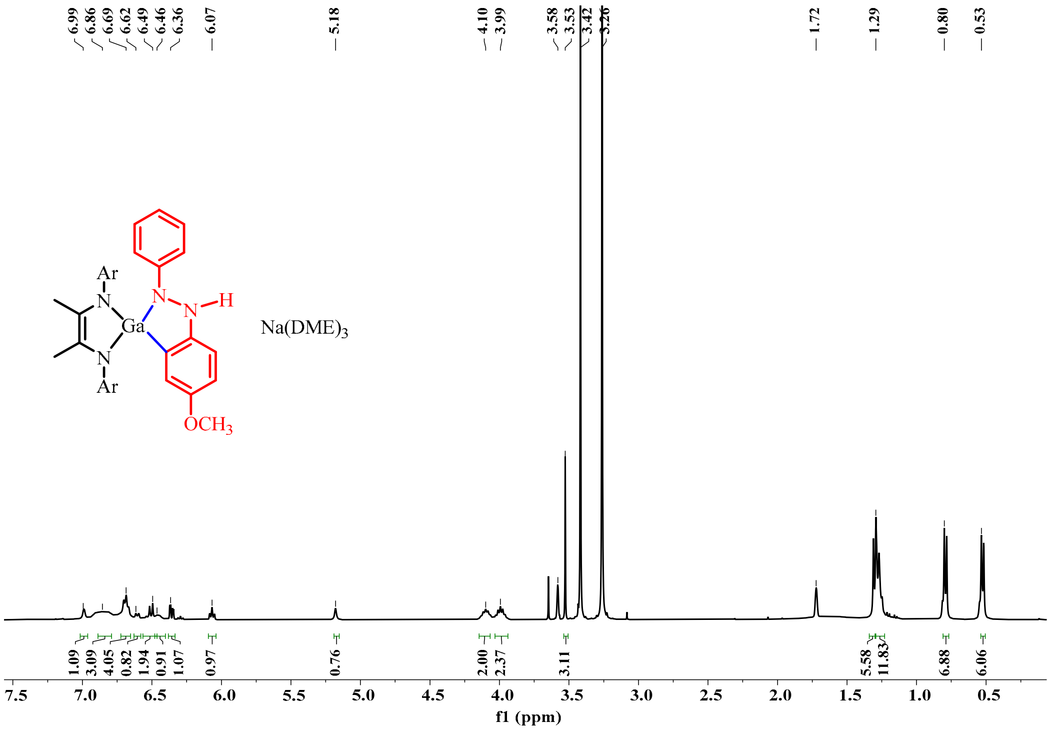

Figure S3. ^{1}H NMR spectrum of **3** (THF-d_8, 298 K).

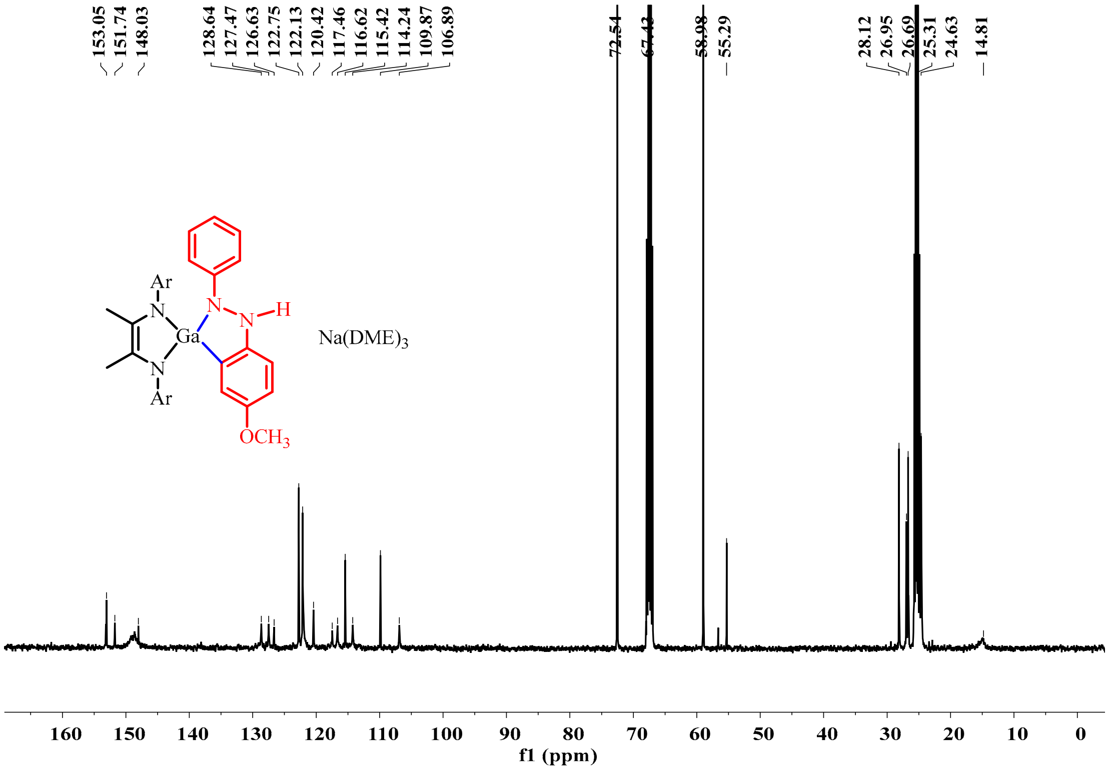

Figure S4. ^{13}C NMR spectrum of **3** (THF-d_8, 298 K).

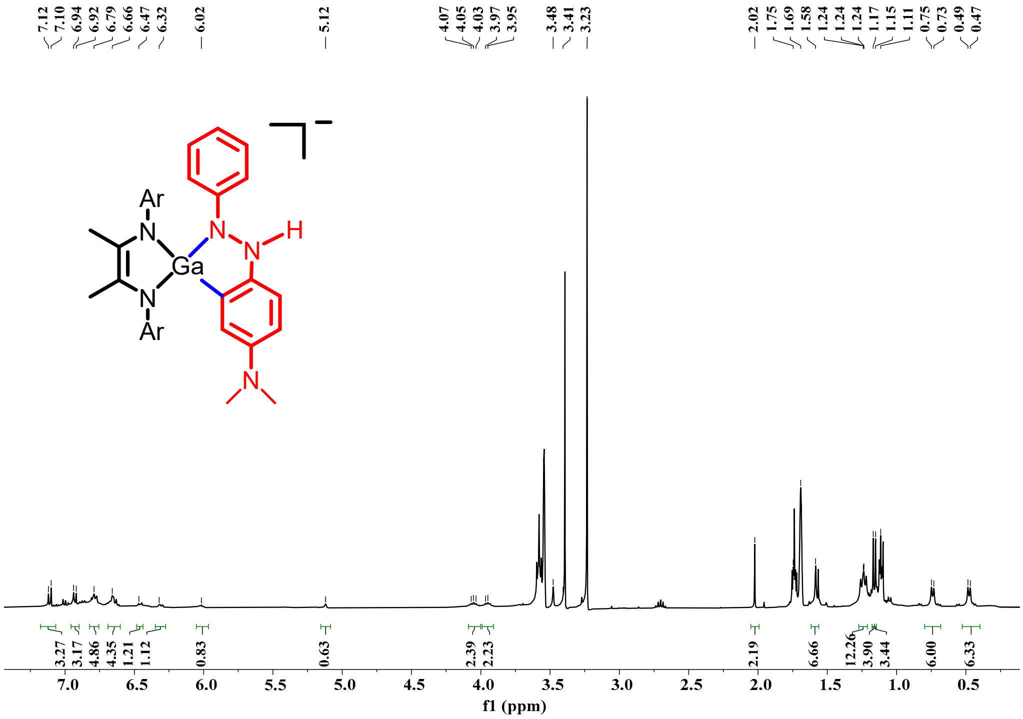

Figure S5. ^{1}H NMR spectrum of **4** (THF-d_8, 298 K).

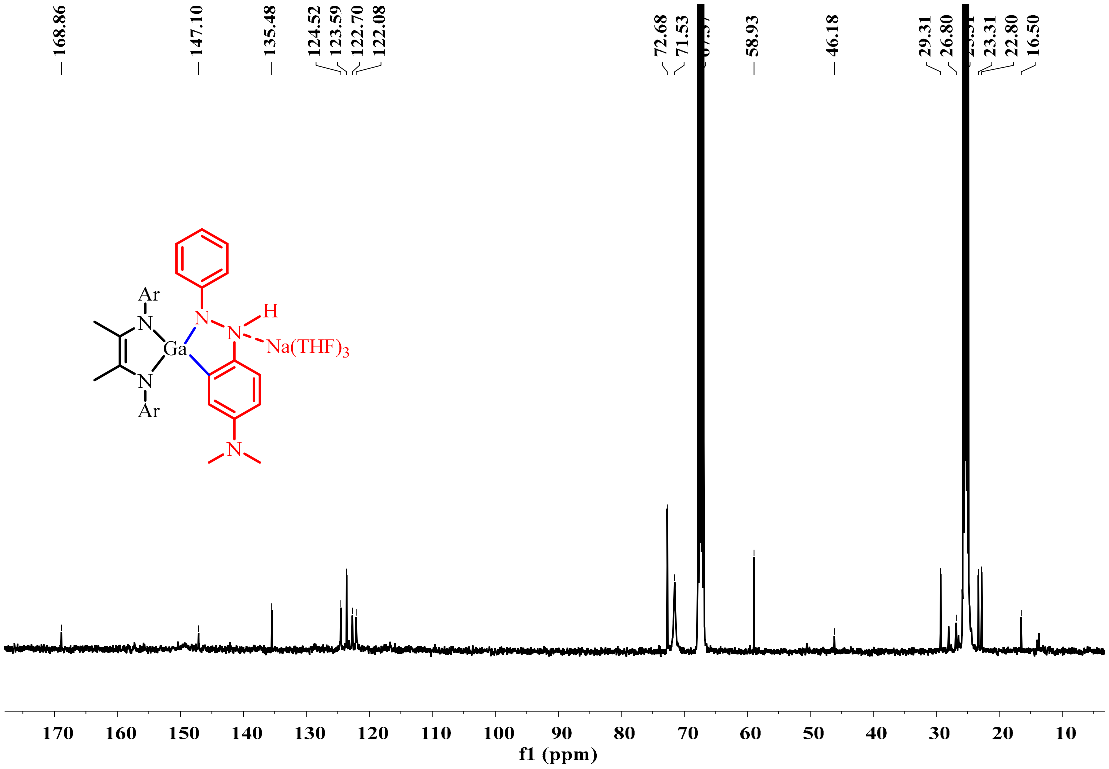

Figure S6. ^{13}C NMR spectrum of **4** (THF-d_8, 298 K).

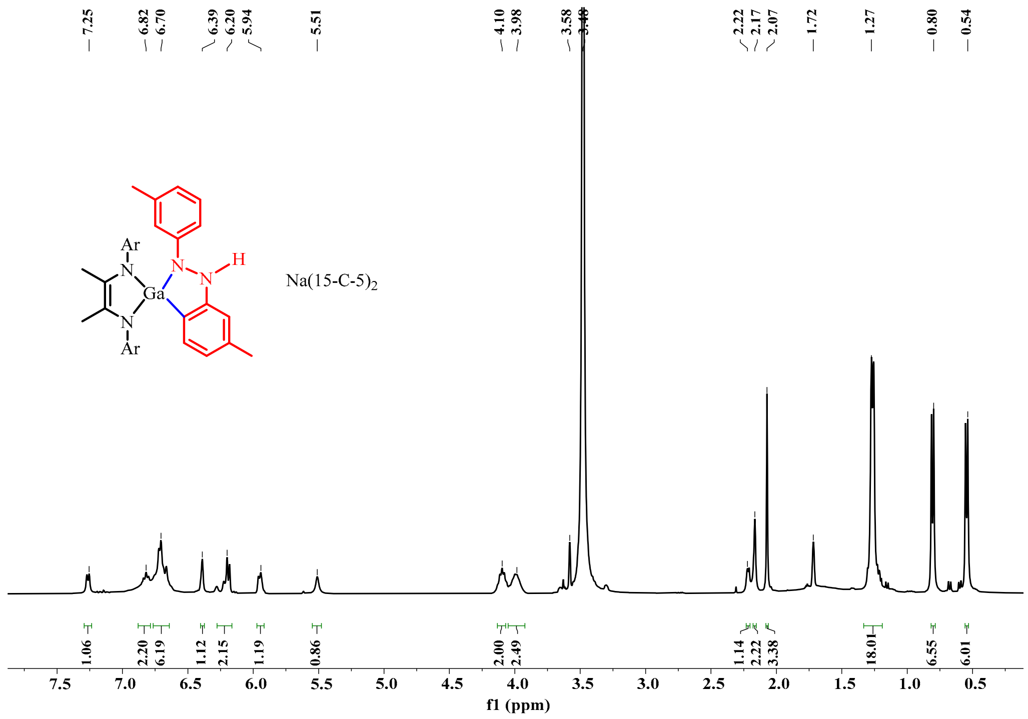

Figure S7. ^{1}H NMR spectrum of **5** (THF-d_8, 298 K).

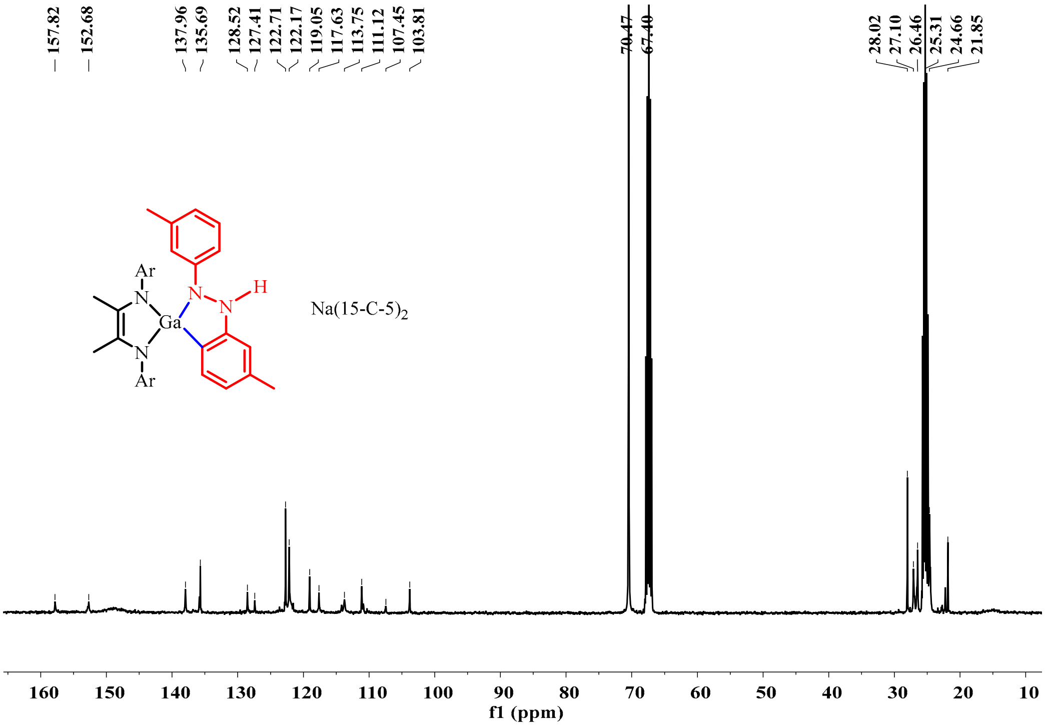

Figure S8. ^{13}C NMR spectrum of **5** (THF-d_8, 298 K).

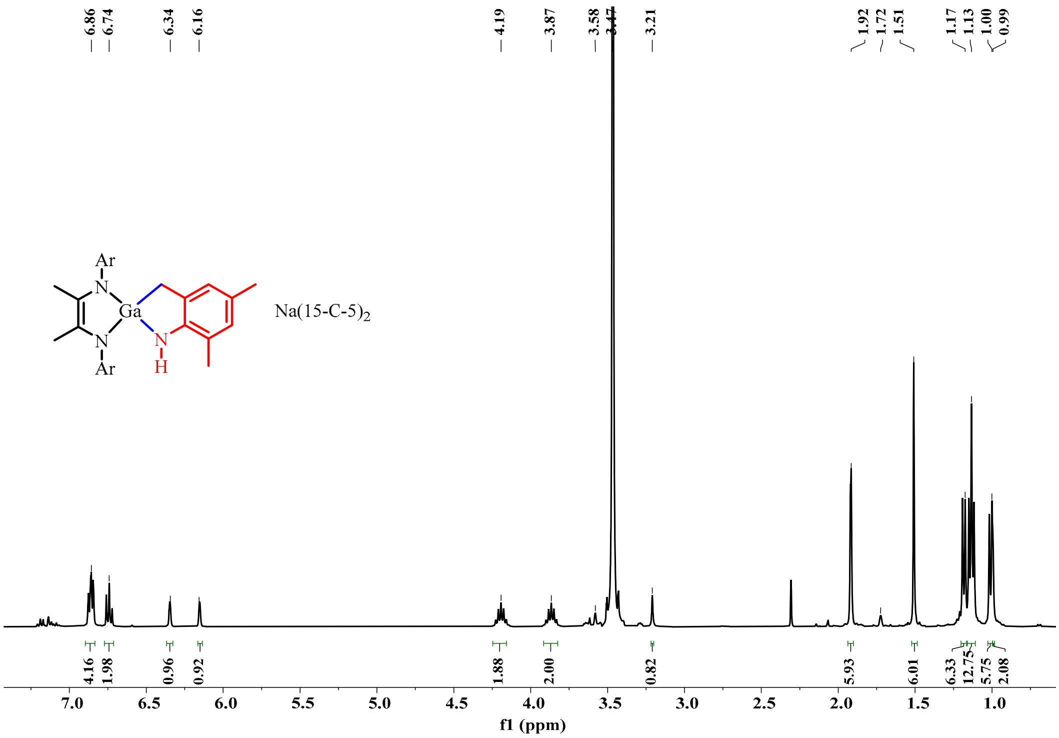

Figure S9. ^{1}H NMR spectrum of **6** (THF-d_8, 298 K).

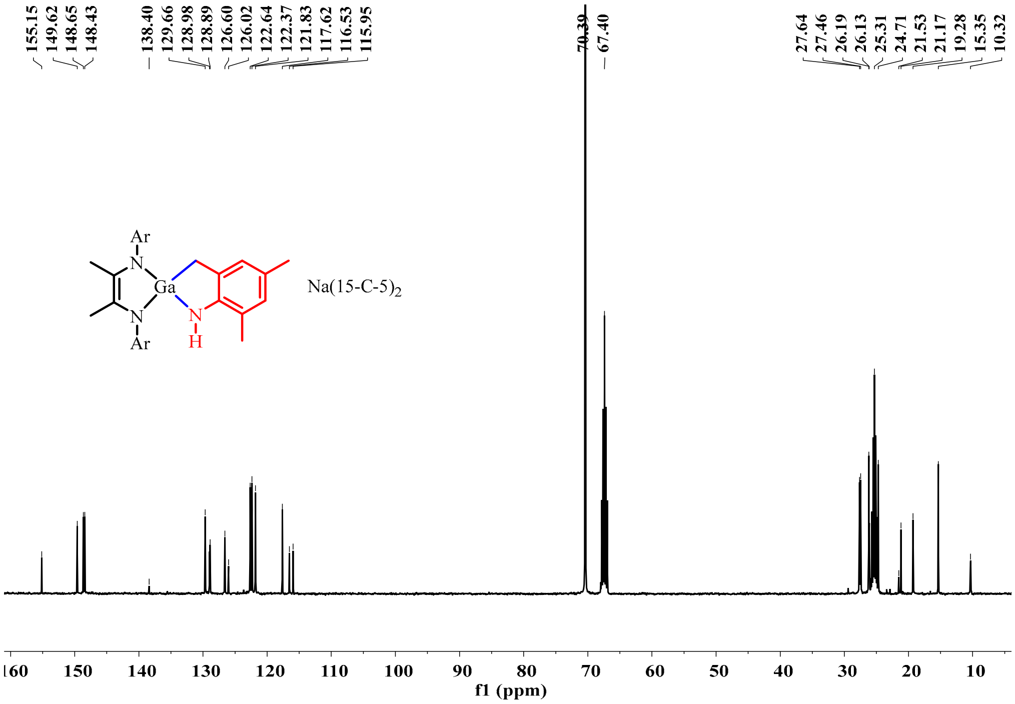

Figure S10. ^{13}C NMR spectrum of **6** (THF-d_8, 298 K).

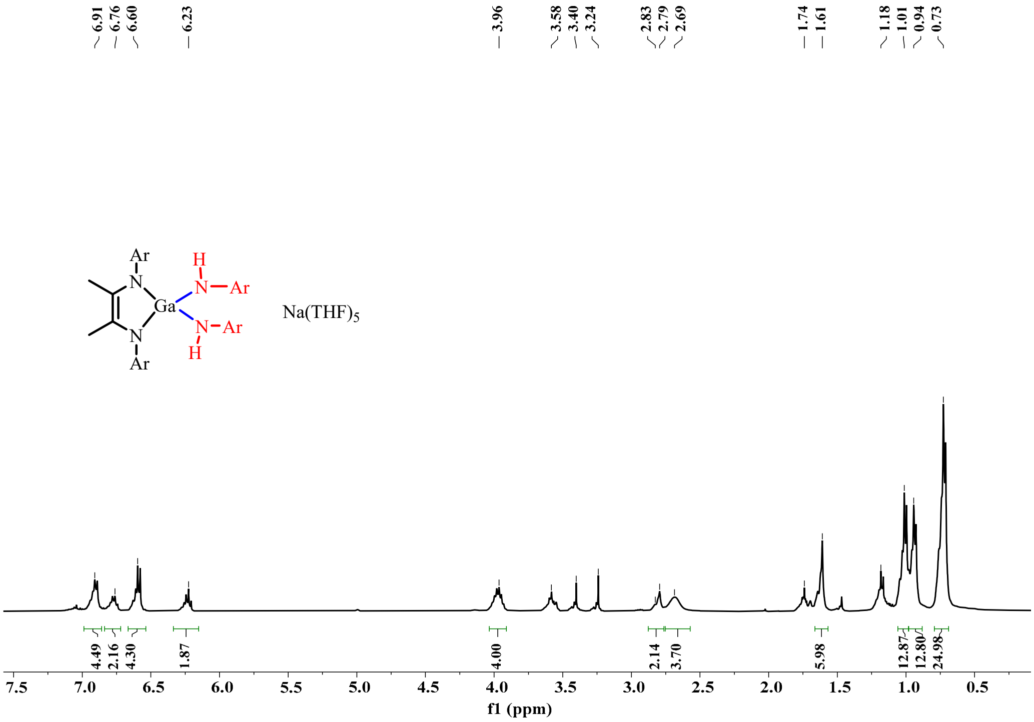

Figure S11. ^{1}H NMR spectrum of **7** (THF-d_8, 298 K).

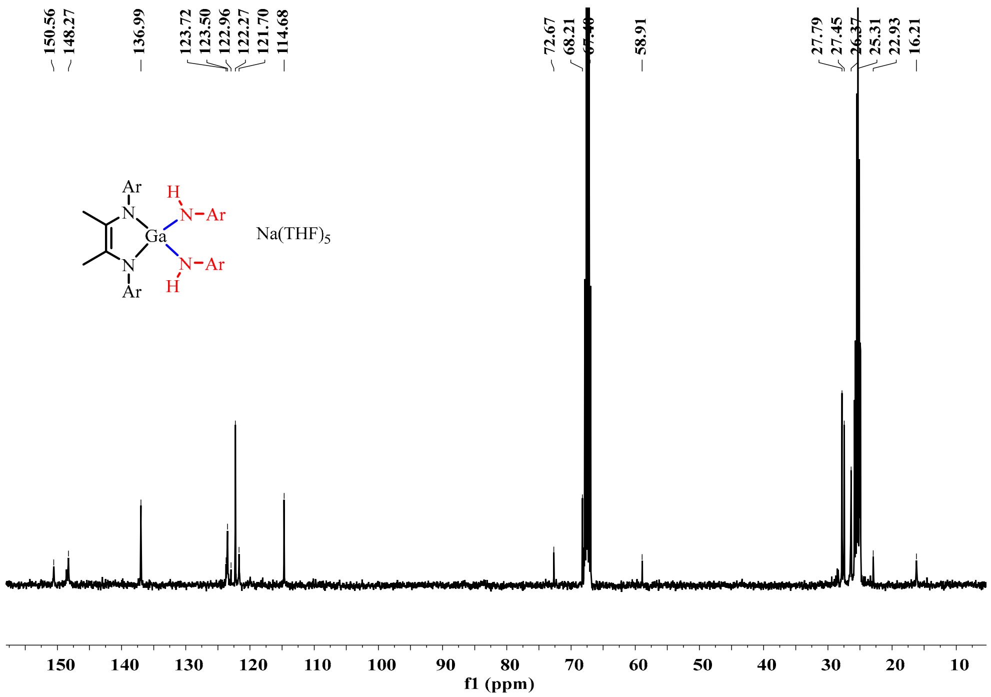

Figure S12. ^{13}C NMR spectrum of **7** (THF-d_8, 298 K).

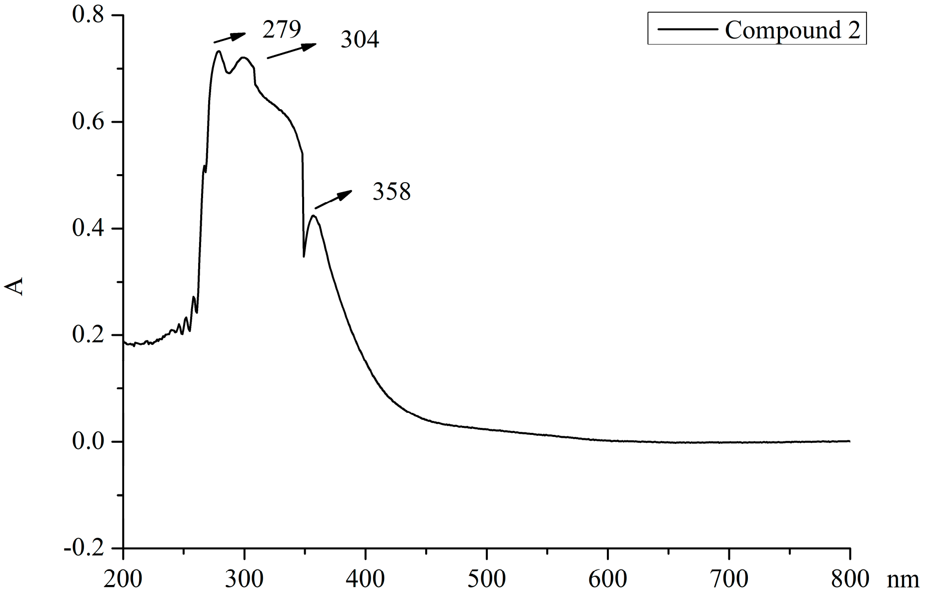

Figure S13. UV−Vis spectra of **2** in THF.

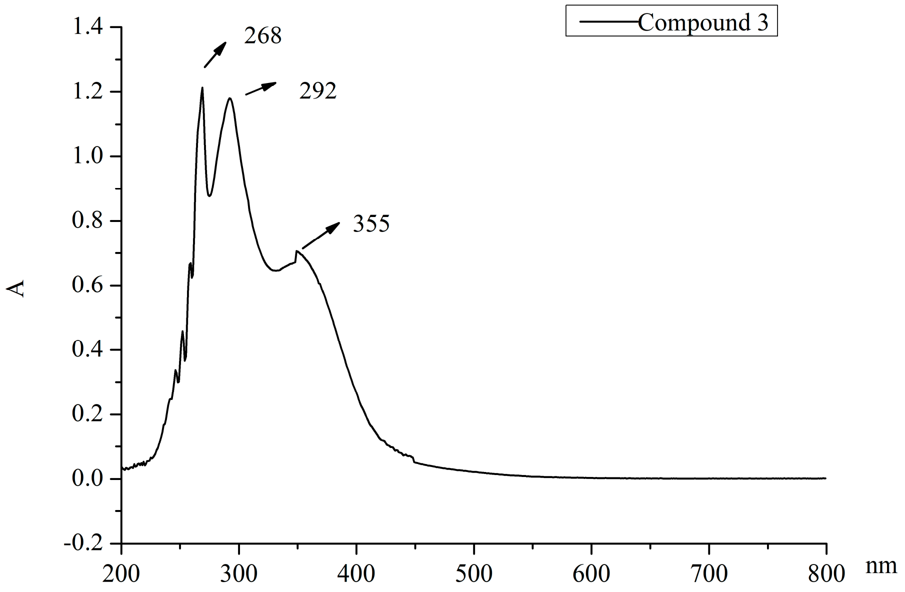

Figure S14. UV−Vis spectra of **3** in THF.

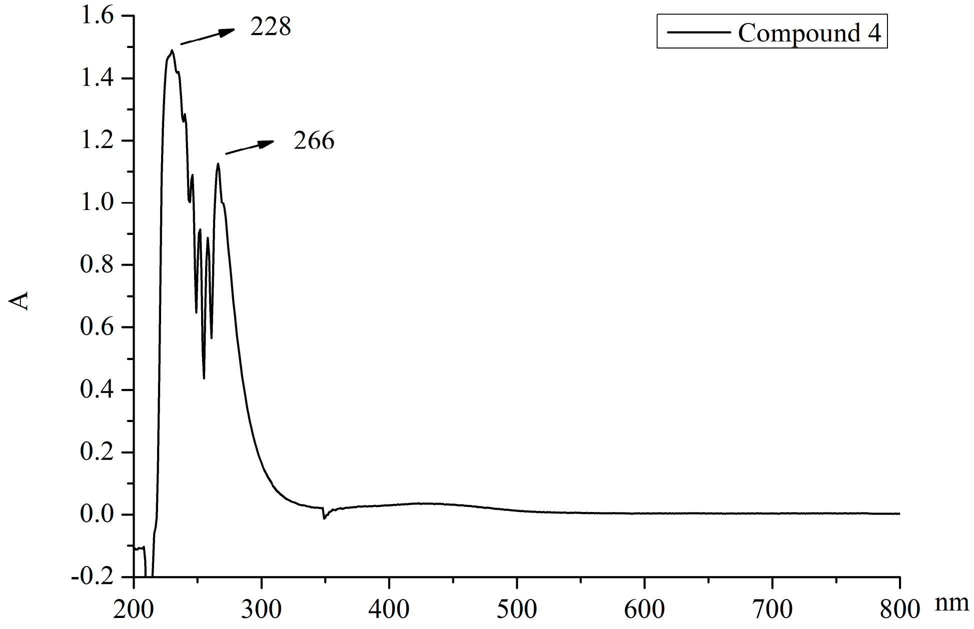

Figure S15. UV−Vis spectra of **4** in THF.

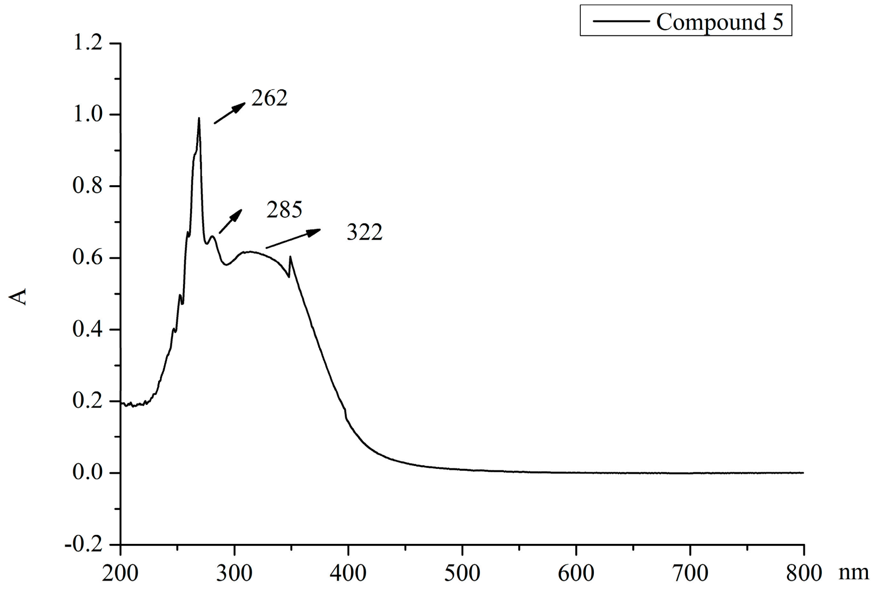

Figure S16. UV−Vis spectra of **5** in THF.

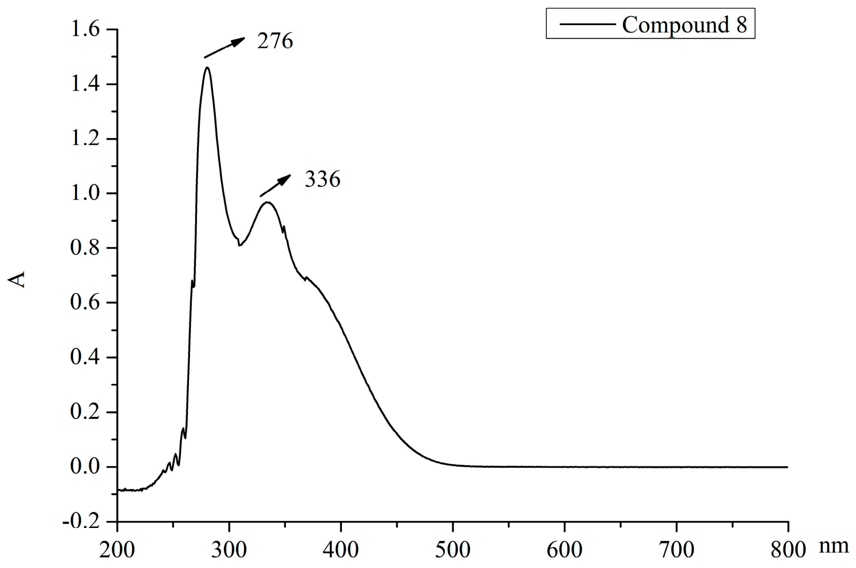

Figure S17. UV−Vis spectra of **6** in THF.

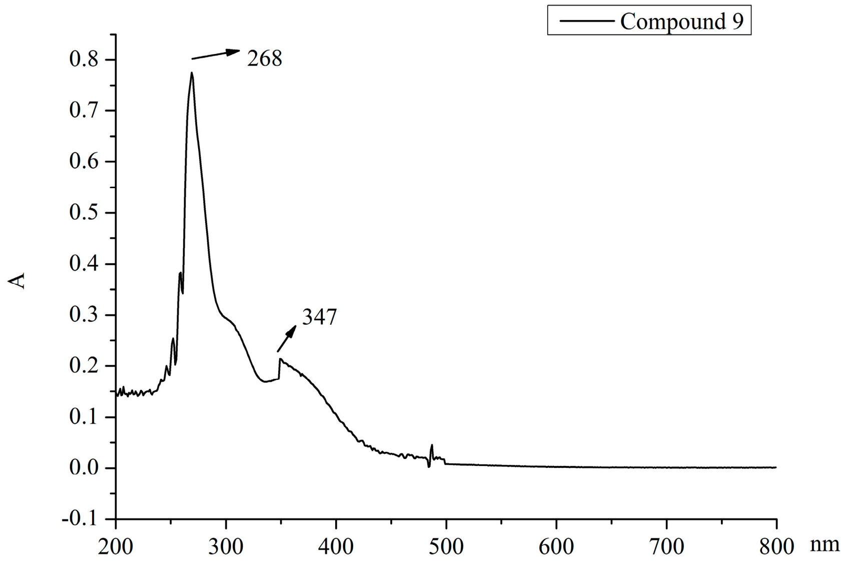

Figure S18. UV−Vis spectra of **7** in THF.

S2. X-ray Crystallographic Analysis

For complexes **2**, **4** and **6**, the X-ray diffraction data were collected with a Bruker APEX-IICCD diffractometer at 150 K (for **2**), 200 K (for **3**), 150 K (for **4**), 210 K (for **5**), 200 K (for **6**), 210 K (for **7**) with graphite-monochromated Ga Kα radiation (for compounds **2**, **4**, λ = 1.34139 Å) and Mo Kα radiation (for compounds **3**, **5**, **6** and **7**, λ = 0.71073). An empirical absorption correction using SADABS was applied for all data.[1] The structures were solved and refined to convergence on F^2 for all independent reflections by the full-matrix least squares method using the SHELXL-2014 programs.[2]

In compound 3, about 0.4 molecules of DME (about 0.1 DME molecules per formula, Z = 4) are co-crystallized, with the corresponding electron density (20 electrons) being removed using the SQUEEZE routine implemented within the software program PLATON,[3] and the resulting .fab

file was processed with OLEX2 1.2[4] using the ABIN instruction. Moreover, some atoms in the DME solvents are disordered and display unusual thermal parameters, which caused the level B alerts. **In compound 5**, Some atoms in the 15-C-5 are disordered and display unusual thermal parameters, which caused the level B alerts. **In compound 6**, about 7.0 molecules of THF (about 1.0 THF molecules per formula, Z = 8) are co-crystallized, with the corresponding electron density (272 electrons) being removed using the SQUEEZE routine implemented within the software program PLATON. Moreover, some atoms in the 15-C-5 are disordered and display unusual thermal parameters, which caused the level B alerts. **In compound 7**, three disordered solvate molecules of THF, which made some the level B alerts. Crystallographic data and refinement details for compounds **2**−**7** are given in Table S1 and S2. CCDC numbers 2349507−2349510, and 2349512, 2349513 (**2**–5 and **6**, **7**). These data can be obtained free of charge from the Cambridge Crystallographic Data Centre www.ccdc.cam.ac.uk/data_request/cif.

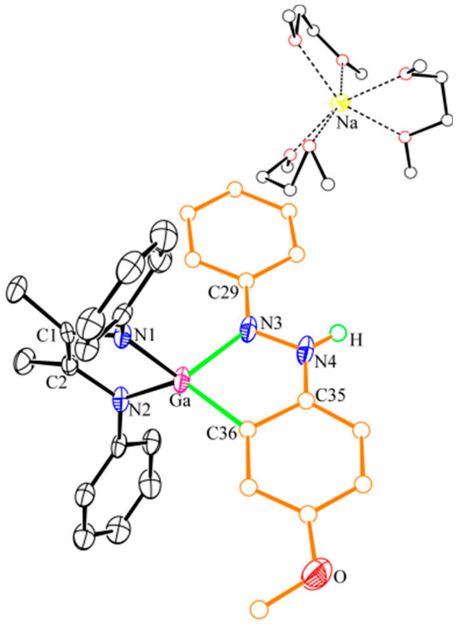

Figure S19. Molecular structure of **3**. Hydrogen atoms and iPr groups have been omitted. The green bonds represent newly formed chemical bonds. Selected bond lengths [Å] and angles: Ga−N1 1.887(8), Ga−N2 1.872(8), N1−C1 1.415(13), N2−C2 1.453(12), C1−C2 1.339(15), Ga−N3 2.026(11), Ga−C36 1.916(13), N3−N4 1.374(12), N3−C29 1.363(18), N4−C35 1.335(14), C35−C36 1.442(18), N4−H 0.8800, N2-Ga-N1 88.7(4), N2-Ga-N3 114.5(4), N2-Ga-C36 126.5(4), N1-Ga-N3 115.4(4), N1-Ga-C36 130.3(5), C36-Ga-N3 83.5(5).

Table S1. Crystallographic data and refinement details for compounds **2–4**..

Compound	**2**	**3**	**4**
formula	$C_{52}H_{80}GaN_4NaO_6$	$C_{53}H_{82}GaN_4NaO_7$	$C_{54}H_{79}GaN_5NaO_3$
M r [g mol -1]	949.91	979.93	938.93
Crystal system	Orthorhombic	monoclinic	monoclinic
Space group	Pna2(1)	C1c1	P2(1)/c

a /Å	20.9145(17)	32.428(6)	17.3299(16)
b /Å	24.200(2)	10.519(2)	14.3240(13)
c /Å	21.3074(8)	21.992(8)	21.249(2)
/°	90	90	90
/°	90	131.793(4)	99.631(3)
/°	90	90	90
V /Å3	10784.4(16)	5593(3)	5200.4(8)
Z	8	4	4
Dcalc/g cm-3	1.170	1.163	1.199
μ [mm -1]	0.769	0.549	0.752
F (000)	4080	2104	2016
θmin /θmax [°]	2.404−52.095	2.738−25.119	3.252−58.627
index ranges	−24 ≤ h ≤ 24,<br>−23 ≤ k ≤ 27,<br>−24 ≤ l ≤ 24	−38≤ h ≤ 38,<br>−12 ≤ k ≤12,<br>−26 ≤ l ≤25	−12≤ h ≤ 22,<br>−15 ≤ k ≤ 17,<br>−26 ≤ l ≤ 26
reflections collected	100254	48159	41939
independent reflections	17493	9674	10547
R_{int}	0.0494	0.0908	0.0461
max/min transmission	0.7505; 0.4946	0.7452; 0.5559	0.7515; 0.4504
data/restraints/ parameters	17493/1/1180	9674/76/581	10547/0/589
GOF on (F^2)	1.176	1.132	1.078
final R indices [I>2σ> (I)]	0.0359/0.0841	0.0967/0.2105	0.0599/0.1384
R indices (all data)	0.0402/0.0860	0.1063/0.2166	0.0653/0.1431
largest diff. peak/hole [e Å $^{-3}$]	0.354/−0.430	1.541/−1.040	0.654/−0.840

Table S2. Crystallographic data and refinement details for compounds **5**−**7**.

Compound	**5**	**6**	**7**
formula	$C_{62}H_{94}GaN_4NaO_{10}$	$C_{57}H_{91}GaN_3NaO_{10}$	$C_{72}H_{116}Ga N_4NaO_5$
M_r [g mol^{-1}]	1148.12	1071.03	1210.39
crystal system	Orthorhombic	monoclinic	monoclinic
space group	P2(1)2(1)2(1)	P121/c1	C12/c1
a [Å]	23.2594(10)	13.082(6)	48.92(10)
b [Å]	25.3580(11)	20.984(7)	12.022(14)
c [Å]	10.8208(4)	45.56(2)	33.36(6)
α[°]	90	90	90
β [°]	90	95.33(3)	132.96(5)
γ[°]	90	90	90

V [Å^3]	6382.2(5)	12453(10)	14357(45)
Z	4	8	8
ρ_{calc}, [g cm^{-3}]	1.195	1.143	1.120
μ [mm^{-1}]	0.495	0.502	0.438
F(000)	2464	4608	5264
$\theta_{min}/\theta_{max}$ [°]	1.829−25.357	1.859−25.052	2.130−25.363
index ranges	$-22 \le h \le 28$, $-30 \le k \le 30$, $-13 \le l \le 12$	$-15 \le h \le 15$, $-25 \le k \le 24$, $-39 \le l \le 54$	$-58 \le h \le 58$, $-14 \le k \le 13$, $-40 \le l \le 40$
reflections collected	31798	97283	96189
independent reflections	11491	21874	13098
R_{int}	0.0811	0.0974	0.0820
max/min transmission	0.7452; 0.6585	0.7452; 0.5578	0.7452;0.6250
data/restraints/ parameters	11491/42/710	21874/30/1310	13098/6/767
GOF on F^2	1.123	1.193	1.024
final R indices [I>2σ (I)]	0.0805/0.1466	0.0775/0.1565	0.0564/0.1451
R indices (all data)	0.1479/0.1723	0.1355/0.1770	0.0891/0.1674
largest diff. peak/hole [e Å^{-3}]	0.908/−0.681	1.762/−0.758	0.701/−0.502

S3. Theoretical Calculations

The model compounds where the 2,6-diisopropylphenyl groups on the nitrogen atoms were replaced by phenyl groups, were used for the products **2−7** in the DFT computations. The structure optimization and NBO bonding analysis for the model compounds **2H−7H** were carried out at the DFT (B3LYP) level with the 6-31G* basis[5,6] sets using the Gaussian 09 program.[7] Geometry optimizations gave bond distances that were in good agreement with the X-ray structures. Bonding analyses were performed by means of natural bond orbital (NBO) analysis and natural population analysis (NPA). Wiberg bond indices (WBI) were evaluated with Weinhold's natural bond orbital method.[8−10]

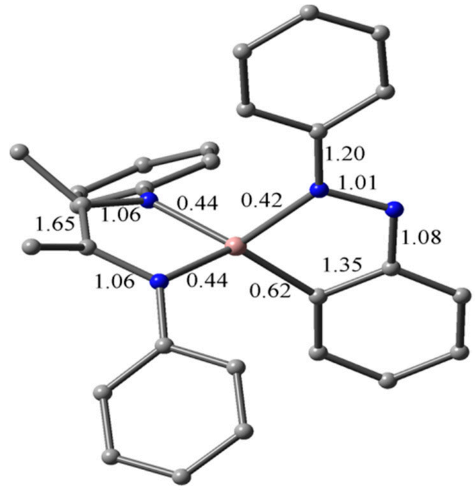

2H

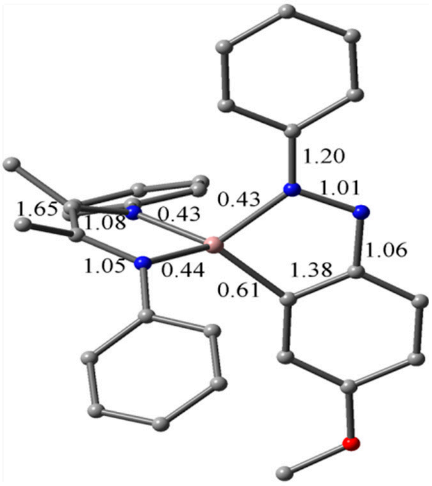

3H

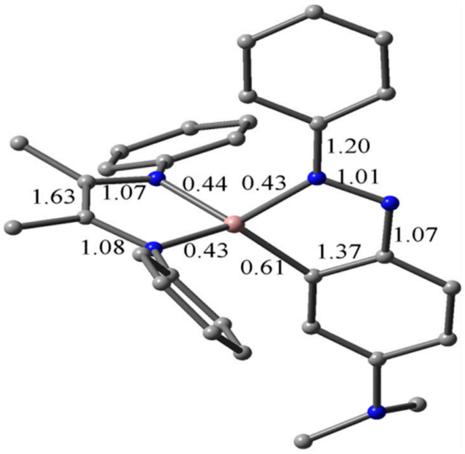

4H

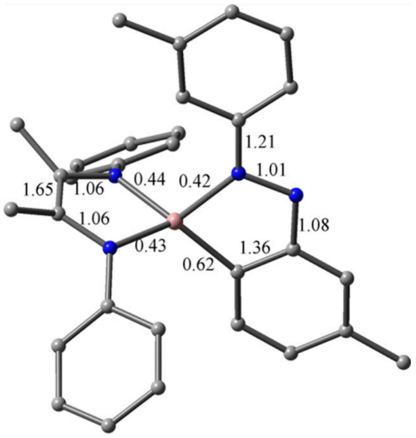

5H

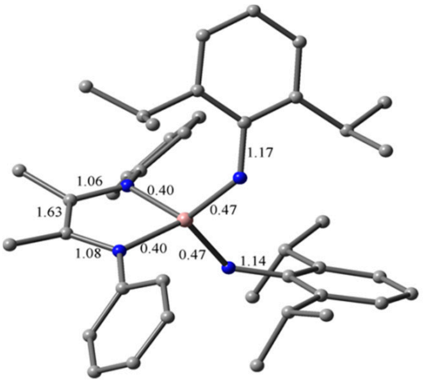

6H

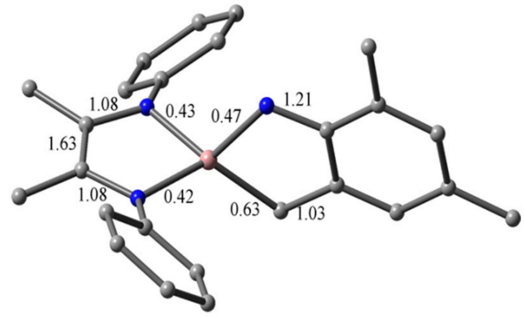

7H

Figure S20. Optimized structures of **2H−7H** labelled with selected bond orders.

Table S3. Natural charges (e) of the model compounds **2H−4H**.

Compound	**2H**	**3H**	**4H**
Ga	1.6555	1.6573	1.6552
L	−1.3905	−1.3909	−1.3856
Small molecule	−1.2650	−1.2665	−1.2696

Table S4. Natural charges (e) of the model compounds **5H−7H**.

Compound	**5H**	**6H**	**7H**
Ga	1.6554	1.6489	1.7738
L	−1.3917	−1.4236	−1.4191
Small molecule	−1.2637	−1.2352	−0.6793, −0.6754

S4. References

1. Sheldrick, G. M. (2016). SADABS v.2016/2, Bruker/Siemens Area Detector Absorption Correction Program, Bruker AXS, Madison, Wisconsin, USA.

2. Sheldrick, G. Crystal structure refinement with SHELXL. *Acta Crystallographica Section C* **2015**, *71*, 3−8.

3. Spek, A. L. PLATON SQUEEZE: a tool for the calculation of the disordered solvent contribution to the calculated structure factors. *Acta Cryst.* **2015**, *C71*, 9–18.

4. Dolomanov, O. V.; Bourhis, L. J.; Gildea, R. J.; Howard, J. A. K.; Puschmann, H. OLEX2: a complete structure solution, refinement and analysis program. *J. Appl. Crystallogr.* **2009**, *42*, 339−341.

5. Becke, A. D. Density-functional thermochemistry. III. The role of exact exchange. *J. Chem. Phys.* **1993,** *98*, 5648−5652.

6. Lee, C.; Yang, W.; Parr, R. G. Development of the Colle-Salvetti correlation-energy formula into a functional of the electron density. *Phys. Rev. B* **1988**, *37*, 785−789.

7. Gaussian 09, Revision C.1, Frisch, M. J.; Trucks, G. W.; Schlegel, H. B.; Scuseria, G. E.; Robb, M. A.; Cheeseman, J. R.; Scalmani, G.; Barone, V.; Mennucci, B.; Petersson, G. A.; Nakatsuji, H.; Caricato, M.; Li, X.; Hratchian, H. P.; Izmaylov, A. F.; Bloino, J.; Zheng, G.; Sonnenberg, J. L.; Hada, M.; Ehara, M.; Toyota, K.; Fukuda, R.; Hasegawa, J.; Ishida, M.; Nakajima, T.; Honda, Y.; Kitao, O.; Nakai, H.; Vreven, T.; Montgomery, Jr., J. A.; Peralta, J. E.; Ogliaro, F.; Bearpark, M.;

Heyd, J. J.; Brothers, E.; Kudin, K. N.; Staroverov, V. N.; Kobayashi, R.; Normand, J.; Raghavachari, K.; Rendell, A.; Burant, J. C.; Iyengar, S. S.; Tomasi, J.; Cossi, M.; Rega, N.; Millam, J. M.; Klene, M.; Knox, J. E.; Cross, J. B.; Bakken, V.; Adamo, C.; Jaramillo, J.; Gomperts, R.; Stratmann, R. E.; Yazyev, O.; Austin, A. J.; Cammi, R.; Pomelli, C.; Ochterski, J. W.; Martin, R. L.; Morokuma, K.; Zakrzewski, V. G.; Voth, G. A.; Salvador, P.; Dannenberg, J. J.; Dapprich, S.; Daniels, A. D.; Farkas, Ö.; Foresman, J. B.; Ortiz, J. V.; Cioslowski, J.; Fox, D. J. Gaussian, Inc., Wallingford CT, **2009**.

8. Sizova, O. V.; Skripnikov, L. V.; Sokolov, A. Y. Symmetry decomposition of quantum chemical bond orders. Journal of Molecular Structure: *THEOCHEM* 2008, *870*, 1−9.

9. Reed, A. E.; Curtiss, L. A.; Weinhold, F. Intermolecular interactions from a natural bond orbital, donor-acceptor viewpoint. *Chem. Rev.* **1988,** *88*, 899−926.

10. Wiberg, K. B. Application of the pople-santry-segal CNDO method to the cyclopropylcarbinyl and cyclobutyl cation and to bicyclobutane. *Tetrahedron* **1968,** *24*, 1083−1096.
